# CD4^+^ T Cells Recognizing PE/PPE Antigens Directly or via Cross Reactivity Are Protective against Pulmonary *Mycobacterium tuberculosis* Infection

**DOI:** 10.1371/journal.ppat.1005770

**Published:** 2016-07-28

**Authors:** Fadel Sayes, Alexandre Pawlik, Wafa Frigui, Matthias I. Gröschel, Samuel Crommelynck, Catherine Fayolle, Felipe Cia, Gregory J. Bancroft, Daria Bottai, Claude Leclerc, Roland Brosch, Laleh Majlessi

**Affiliations:** 1 Institut Pasteur, Unité de Pathogénomique Mycobactérienne Intégrée, Paris, France; 2 Institut Pasteur, Unité de Régulation Immunitaire et Vaccinologie, Paris, France; 3 INSERM U1041, Paris, France; 4 London School of Hygiene and Tropical Medicine, London, United Kingdom; 5 University of Pisa, Ricerca Traslazionale e delle Nuove Tecnologie in Medicina e Chirurgia, Pisa, Italy; Portland VA Medical Center, Oregon Health and Science University, UNITED STATES

## Abstract

*Mycobacterium tuberculosis* (*Mtb*), possesses at least three type VII secretion systems, ESX-1, -3 and -5 that are actively involved in pathogenesis and host-pathogen interaction. We recently showed that an attenuated *Mtb* vaccine candidate *(Mtb* Δ*ppe25-pe19)*, which lacks the characteristic ESX-5-associated *pe/ppe* genes, but harbors all other components of the ESX-5 system, induces CD4^+^ T-cell immune responses against non-*esx-5*-associated PE/PPE protein homologs. These T cells strongly cross-recognize the missing *esx-5*-associated PE/PPE proteins. Here, we characterized the fine composition of the functional cross-reactive Th1 effector subsets specific to the shared PE/PPE epitopes in mice immunized with the *Mtb* Δ*ppe25-pe19* vaccine candidate. We provide evidence that the *Mtb* Δ*ppe25-pe19* strain, despite its significant attenuation, is comparable to the WT *Mtb* strain with regard to: (i) its antigenic repertoire related to the different ESX systems, (ii) the induced Th1 effector subset composition, (iii) the differentiation status of the Th1 cells induced, and (iv) its particular features at stimulating the innate immune response. Indeed, we found significant contribution of PE/PPE-specific Th1 effector cells in the protective immunity against pulmonary *Mtb* infection. These results offer detailed insights into the immune mechanisms underlying the remarkable protective efficacy of the live attenuated *Mtb* Δ*ppe25-pe19* vaccine candidate, as well as the specific potential of PE/PPE proteins as protective immunogens.

## Author Summary

*Mycobacterium tuberculosis* (*Mtb*), the causative agent of human tuberculosis, is one of the most widely spread human pathogens, responsible for more than 9.6 million of new tuberculosis cases and 1.5 million deaths, annually. The resurgence of pulmonary tuberculosis in immuno-compromised patients, including HIV-co-infected populations, and increasing spread of drug-resistant *Mtb* strains are worrying. Given the estimated 2 billion cases of latent *Mtb* infections and the only partial efficacy of the unique, currently available tuberculosis-vaccine *Mycobacterium bovis* BCG (Bacille Calmette-Guerin) it is necessary to develop improved vaccines. Here, we demonstrate that the host cellular immunity, mediated by CD4^+^ T lymphocytes, specific to the “PE/PPE” families of mycobacterial antigens, contribute to the protection against *Mtb*-induced disease. We revealed the fine composition of the PE/PPE-specific T cells by characterizing their effector functions and differentiation status. We previously described a live attenuated mycobacterial strain as a vaccine candidate that is able to induce such CD4^+^ T cells and which displays particular properties at stimulating the cells of the innate immune system. These responses play a central role in the initiation of the host defense and in the protection against tuberculosis. Our results pave the way for further development of candidates in preclinical models of anti-tuberculosis vaccination.

## Introduction

It is estimated that two billion people are latently infected with *Mycobacterium tuberculosis* (*Mtb)*, and this huge reservoir is sustaining the pan/epidemic spread of the bacterium. Due to the relative inefficiency of the *Mycobacterium bovis* BCG (Bacille Calmette-Guerin) vaccine in preventing these latent infections becoming active tuberculosis (TB) disease cases in adolescents and adults, new improved TB vaccines are warranted [1].

*Mtb* harbors five chromosomal *esx* clusters of highly conserved genes, which code for specialized type VII secretion systems (T7SSs), some of which are also conserved in other mycobacteria [2]. Some of these systems are dedicated to the export/secretion of key mycobacterial factors and play a determinant role in host-pathogen interaction [3]. Several lines of evidence indicate that the construction of genetically modified mycobacterial strains expressing WT and/or mutated variants of these specialized T7SSs is a promising strategy to set up new live attenuated TB vaccines [4–7]. We have previously shown that BCG complemented with the *esx-1* gene cluster (BCG::ESX-1), produces and secretes the 6 kDa-Early Secreted Antigenic Target (ESAT-6, EsxA) and its partner, the 10 kDa-Culture Filtrate Protein (CFP-10, EsxB) thereby inducing specific host immune responses (S1A and S1B Fig) that ultimately confer improved protection against an *Mtb* challenge in animal models, relative to the parental BCG strain [4, 8, 9]. Sweeny and colleagues generated a recombinant *Mycobacterium smegmatis* strain harboring the orthologous *Mtb esx-3* region, which displays an improved protective efficacy compared to BCG [6]. More recently, we developed an attenuated *Mtb esx-5* mutant, i.e., *Mtb* Δ*ppe25-pe19*, lacking the five *esx-5*-coded *pe/ppe* genes, as a promising vaccine candidate [5, 10].

Most of the *esx* loci contain clusters of genes coding for members of the PE/PPE protein families. These proteins are named after their characteristic N-terminal Pro-Glu (PE) or Pro-Pro-Glu (PPE) motifs and are unique to the mycobacterial species. The genome of the *Mtb* H37Rv strain contains 99 *pe* and 69 *ppe* genes, which most probably evolved from ancestral *esx*-associated *pe/ppe* genes [11]. Although the function of most PE/PPE proteins remains to be unraveled [12–14], some of them have been demonstrated to play a role in mycobacterial virulence, being involved in mycobacterial growth in macrophages and/or in the mouse infection model, or in modulation of mycobacteria-mediated inhibition of phagosome maturation [10, 15–21]. PE/PPE proteins display numerous repetitive sequences and possess abundant immunogenic regions, representing a rich source of B and T cell epitopes [22].

The *esx-5* region of *Mtb* (*rv1782-rv1798*) harbors 2 *pe* (*pe18*, *19*) and 3 *ppe* (*ppe25*, *26*, *27*) genes (S1A Fig). The corresponding PE18, 19, and PPE25, 26, 27 proteins are exported/secreted through the transmembrane channel of the ESX-5 secretion apparatus, involving the ESX-Conserved Component EccD_5_ [10] (S1B Fig, right). In addition, many other non-*esx-5*-associated PE/PPE proteins with various degrees of sequence similarity with their *esx-5*-coded homologs, are also exported/secreted via the ESX-5 system [3, 23, 24].

Our recent observation that the *Mtb* Δ*ppe25-pe19* strain, devoid of the five *esx-5*-coded *pe/ppe* genes, is attenuated for growth in immune-competent and SCID mice, indicates a role in virulence of these PE/PPE proteins [5, 10]. Importantly, as the transmembrane channel EccD_5_ is unaffected/intact, the *Mtb* Δ*ppe25-pe19* strain continues to be able to export PE/PPE proteins, which are encoded outside the *esx-5* locus. As a results, IFN-γ^+^ CD4^+^ T-cell responses are induced against a plethora of non-*esx-5*-coded PE/PPE homologs in the immunized host. The involved T cells, via their high cross-reactivity, recognize *esx-5*-coded PE/PPE virulence-related factors (S1B Fig, right). Therefore, due to the expression of a functional EccD_5_-associated transmembrane channel and an intact ESX-5 T7SS, the *Mtb* Δ*ppe25-pe19* strain shows the unique property to induce cross-reactive T-cell immunity against the *esx-5-*associated PE/PPE virulence-related factors, despite their absence in this strain [3, 5]. Epitope mapping of the PE/PPE proteins in bovines also revealed that the highly immunogenic nature of PE/PPE immunogens is essentially driven by a substantial degree of cross-reactivities in the elicited T cells, which results from the sequence homologies among the PE/PPE proteins [25, 26]. An *Mtb eccD*_*5*_ KO strain, largely deficient in PE/PPE protein secretion, does not phenocopy the *Mtb* Δ*ppe25-pe19* strain and is markedly less protective in vaccination assays performed in the mouse model [5]. This observation strongly suggests that immunity to PE/PPE proteins is a relevant requisite for an efficient protection against TB.

The distribution of the functional CD4^+^ T-cell subsets defines the quality of the adaptive immune response in infectious diseases including TB [27] and several reports indicate that, at least in animal preclinical models, poly-functional CD4^+^ T cells mediate protection [28]. Therefore, we here characterize at the single-cell level the functionality and some aspects of differentiation status of the cross-reactive PE/PPE-specific Th1 cells induced by *Mtb* Δ*ppe25-pe19* immunization and evaluated the contribution of PE/PPE-specific T cells in the protective immunity against pulmonary *Mtb* infection in mice. These experiments provided new insights on the potential of PE/PPE proteins as protective immunogens. Moreover, the *Mtb* Δ*ppe25-pe19* mutant is able to secrete ESX-1 substrates and thereby elicits CD4^+^ T-cell responses against these protective immunogens. In addition to its particular T-cell antigenicity, the *Mtb* Δ*ppe25-pe19* exhibits unique properties to trigger the host innate immunity. Unlike BCG, the expression of a functionally active ESX-1 system enables the *Mtb* Δ*ppe25-pe19* vaccine candidate to induce phagosomal membrane rupture and thereby establishing a phagosome-cytosol communication inside phagocytes, a phenomenon which has instrumental consequences on the activation of innate immunity [29–32]. These results elucidate part of the immune properties of the remarkable protective capacity of the live attenuated *Mtb* Δ*ppe25-pe19* vaccine candidate.

## Results

### Fine composition of PE/PPE-specific Th1 functional subsets induced by *Mtb* Δ*ppe25-pe19* immunization

We previously identified two groups of PPE25- and PE19-derived MHC-II (I-A^b^)-restricted T-cell epitopes. One group is highly specific to *Mtb esx-5*-encoded PE/PPE proteins and show no homologies with other PE/PPE (S1A Table), while the second group contains epitopes which are shared with PE/PPE homologs coded outside of *esx-5* (S1B Table) [5]. Immunization of C57BL/6 (H-2^b^) mice with the attenuated *Mtb* Δ*ppe25-pe19* strain confirmed and extended our previous finding that this strain is unable to induce Th1 immunity, i.e., IL-2, TNF-α, and IFN-γ responses, against the *esx-5*-specific PE/PPE epitopes (S2 Fig). However, this attenuated vaccine candidate preserved its capacity to induce Th1 immunity against the PE/PPE homologs coded outside of *esx-5*, due to the expression of a functional ESX-5 transmembrane channel associated to EccD_5_ [33, 34]. Such cross-reactive Th1 cells specific to the shared PE/PPE epitopes recognize the ESX-5-associated and virulence-related PE/PPE proteins, which are absent in the *Mtb* Δ*ppe25-pe19* strain.

**Fig 1:**
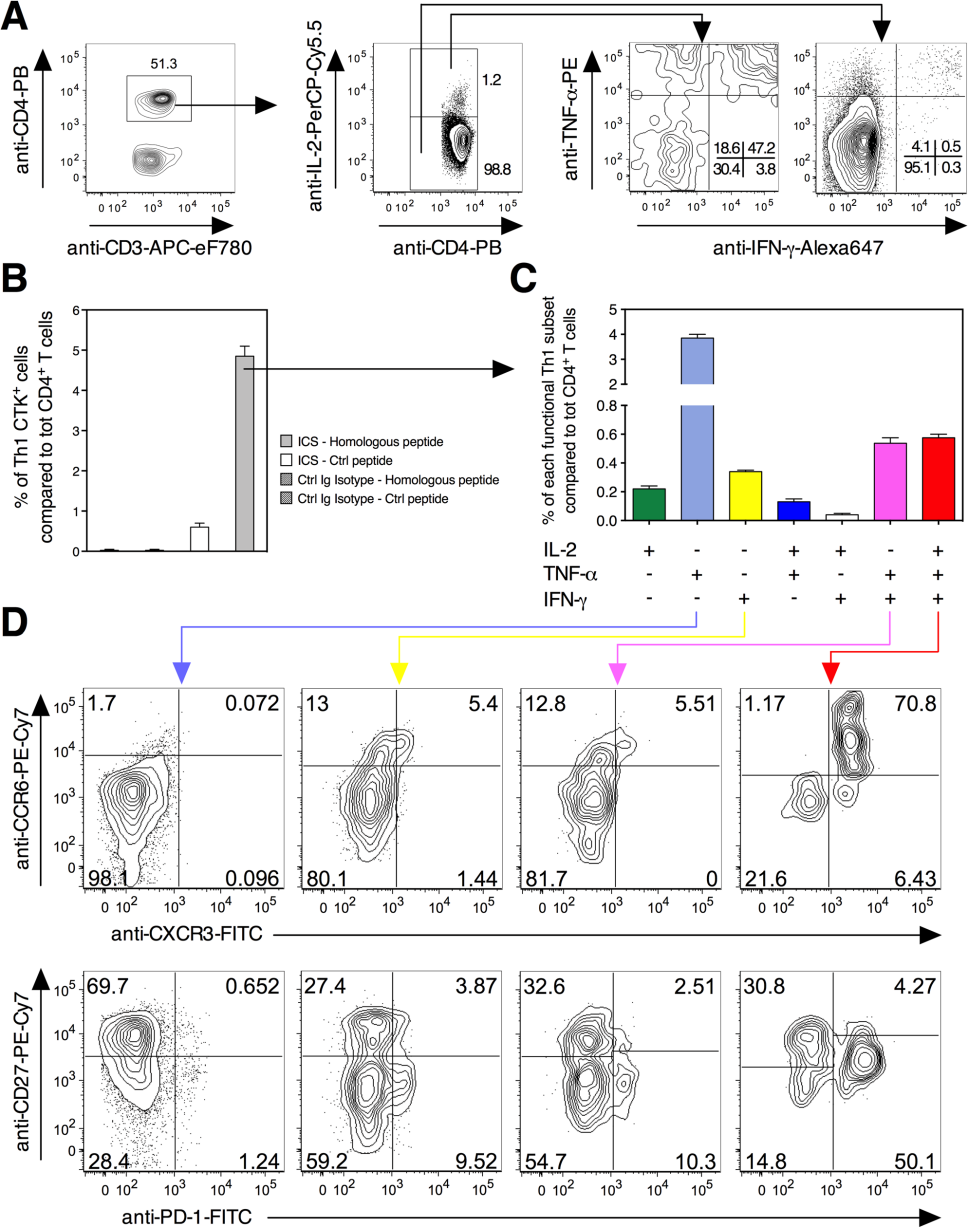
Cytometric strategy used to identify different functional Th1 subsets specific to mycobacterial antigens. A) Splenocytes from C57BL/6 mice (*n* = 3 per group), injected s.c. with 1 x 10^6^ CFU/mouse of *Mtb* Δ*ppe25-pe19*, were stimulated *in vitro* with the PPE25:1–20 peptide at 4 weeks p.i., prior to surface and intracellular staining to detect single, double or triple positive antigen-specific Th1 cells. B) Percentage of cells producing any of the Th1 IL-2/TNF-α/IFN-γ cytokines (CTK) compared to total CD4^+^ T splenocytes. C) Definition of antigen-specific Th1 effector subsets as a function of their IL-2/TNF-α/IFN-γ expression and their percentages compared to total CD4^+^ T splenocytes. Means ± SD are standard deviations. D) Surface expression of CCR6, CXCR3, CD27 and PD-1 as analyzable in TNF-α^+^ or IFN-γ^+^ single positive, TNF-α^+^ IFN-γ^+^ double positive or IL-2^+^ TNF-α^+^ IFN-γ^+^ triple positive, antigen-specific functional Th1 subsets. Data are representative of two independent experiments. See furthermore S3 Fig.

To delineate the effector mechanisms of PE/PPE-specific T-cell immunity, we next subjected such T-cell responses to a fine analysis of the functional CD3^+^ CD4^+^ Th1 subsets by IL-2-, TNF-α-, and IFN-γ-specific IntraCellular Staining (ICS). We first set up the strategy for the PPE25:1-20-specific responses in the spleen of *Mtb* Δ*ppe25-pe19*-immunized mice (Figs 1A and S3A and S3B). This shared epitope is representative of the identified PE/PPE peptides listed in the S1 Table. This approach allowed the determination of the frequencies of total antigen specific Th1 cytokine-producing cells (Fig 1B), as well as the definition of seven functional subsets, which are single, double, or triple positive for the expression of these key Th1 cytokines and their percentages compared to total CD4^+^ T cells (Fig 1C). Moreover, as (i) human *Mtb*-specific memory CD4^+^ T cells are enriched in the T-cell population expressing the chemokine receptors CCR6 and CXCR3 [35, 36], (ii) the Programmed Cell Death-1 (PD-1) marker is associated with proliferative potential, self maintenance, IFN-γ production and protection in the context of anti-mycobacterial immunity [37, 38], and (iii) CD27 expression is a pertinent marker to distinguish different Th1 effector subsets [39], we also performed, together with ICS, simultaneous surface staining with these markers in order to characterize the differentiation status of the antigen-specific, functional Th1 subsets whose the numbers were high enough to allow such analysis (Fig 1D). For instance, in this framework, most of the TNF-α^+^ single positive cells were CCR6^-^ CXCR3^-^ and PD1^-^, only a few percentages of IFN-γ^+^ single positive or TNF-α^+^ IFN-γ^+^ double positive cells were CCR6^+^ CXCR3^+^ and CD27^-^ PD1^+^, while the triple positive Th1 cells contained the highest percentages of CCR6^+^ CXCR3^+^ and CD27^-^ PD-1^+^ cells.

**Fig 2:**
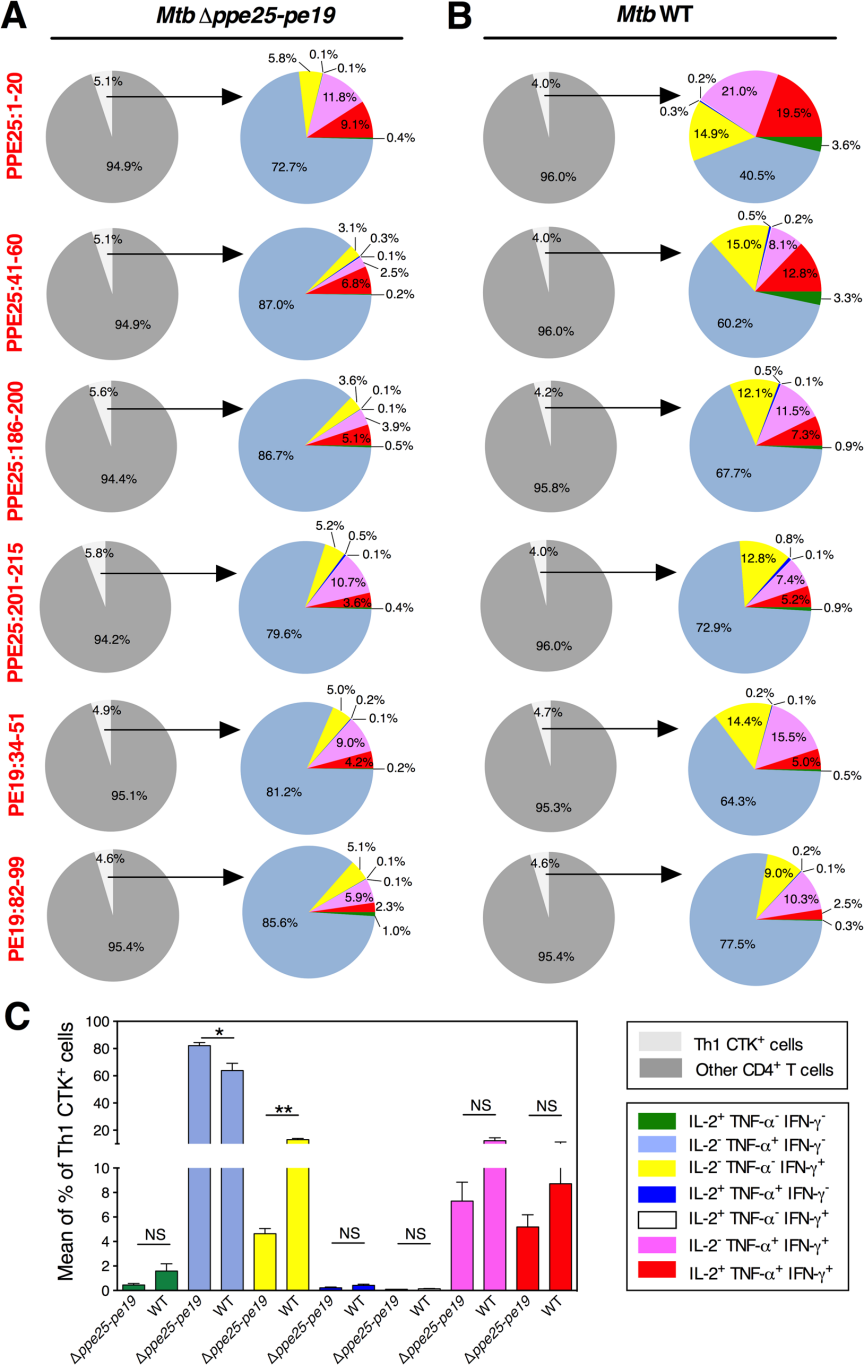
Comparative study of the functional Th1 effector subsets specific to shared PE/PPE epitopes subsequent to immunization with the *Mtb* Δ*ppe25-pe19* or WT strain. Frequencies of different Th1 cytokine-producing splenic CD4^+^ T effectors, at 4 weeks p.i., in C57BL/6 mice (*n* = 5 per group) injected s.c. with 1 x 10^6^ CFU/mouse of the *Mtb* Δ*ppe25-pe19* (A) or the *Mtb* WT strain (B) and stimulated *in vitro* with 10 μg/ml of individual shared PPE25 and PE19 peptides. C) Means ± SD of the frequencies of each Th1 subset, as cumulated for all the studied shared peptides, compared between the *Mtb* Δ*ppe25-pe19*- and the *Mtb* WT-immunized groups. NS = not significant, *, ** = statistically significant, as determined by Mann-Whitney test, *p*<0.05 or *p*<0.005, respectively. The results are representative of two independent experiments. See furthermore S4 Fig.

To get mechanistic insights towards the fine composition of functional Th1 cells generated subsequent to vaccination with *Mtb* Δ*ppe25-pe19*, we first performed such detailed comparative analyses in the groups of *Mtb* Δ*ppe25-pe19*- or *Mtb* WT-immunized mice (Fig 2A and 2B). We observed that the profile of the Th1 responses specific to the different shared PE/PPE epitopes studied was overall similar. In the *Mtb* Δ*ppe25-pe19*-immunized mice, different degrees of PE/PPE-specific functional T subsets were present, which ranged from very small percentages of IL-2^+^ TNF-α^-^ IFN-γ^-^ (green), predominant amounts of IL-2^-^ TNF-α^+^ IFN-γ^-^ (blue) to intermediate percentages of IL-2^-^ TNF-α^-^ IFN-γ^+^ (yellow) single positive Th1 cells. Moreover, IL-2^+^ TNF-α^+^ IFN-γ^-^ (dark blue) and IL-2^+^ TNF-α^-^ IFN-γ^+^ (white) double positive Th1 cells were barely found, while intermediary levels of IL-2^-^ TNF-α^+^ IFN-γ^+^ (purple) double positive and IL-2^+^ TNF-α^+^ IFN-γ^+^ (red) triple positive Th1 subsets were detected. In these mice, the cumulated numbers of IL-2^-^ TNF-α^+^ IFN-γ^+^ or IL-2^+^ TNF-α^+^ IFN-γ^+^ cells specific to the totality of the shared PE/PPE epitopes can be estimated at 7.6 x 10^5^ and 5.4 x 10^5^ splenocytes per mouse, respectively.

**Fig 3:**
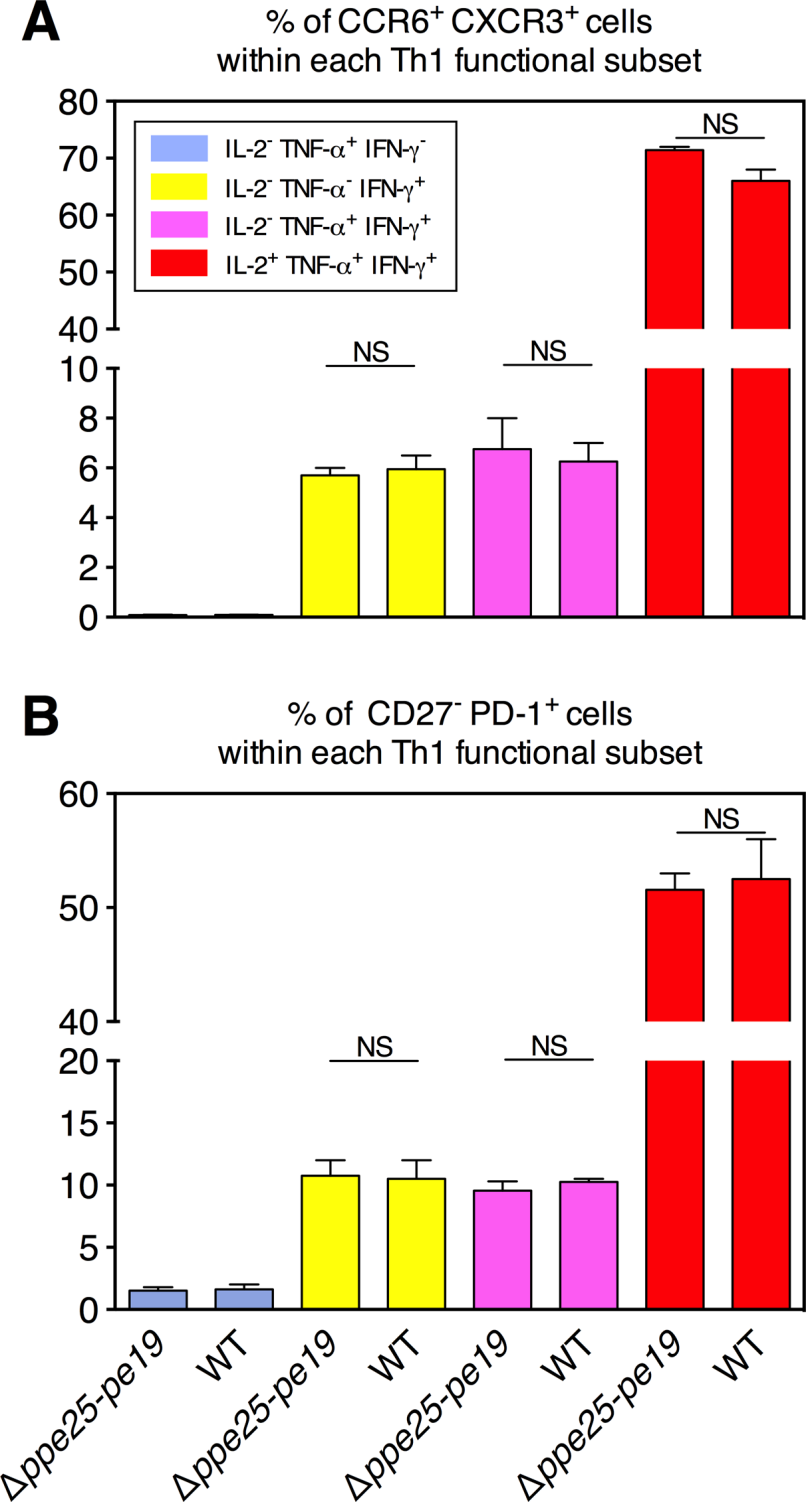
Comparative study of the differentiation status of the antigen-specific functional Th1 subsets in *Mtb* Δ*ppe25-pe19-* or *Mtb* WT-immunized mice. Splenocytes from the immunized mice were stimulated with the representative PPE25:1–20 synthetic peptide as described in Materials and Methods, stained for the surface differentiation markers, and then processed for ICS specific to Th1 cytokines. Percentages of CXCR3^+^ CCR6^+^ (A) or PD-1^+^ CD27^-^ (B) cells were determined, as detailed in the Fig 1D, subsequent to gating on different functional Th1 subsets. Results are means ± SD of experimental duplicates.

Comparison with the *Mtb* WT-immunized mice (Fig 2B) showed that the composition of the functional effector Th1 subsets specific to different shared PE/PPE epitopes were globally similar to those found in the *Mtb* Δ*ppe25-pe19*-immunized group, except for the frequencies of the terminally differentiated IL-2^-^ TNF-α^+^ IFN-γ^-^ (blue) cells, which decreased for the benefit of the terminally differentiated IL-2^-^ TNF-α^-^ IFN-γ^+^ (yellow) population (Fig 2C). This finding suggests that the virulence/persistence of WT *Mtb* might fine-tune such functional switches. As expected, no such Th1 subsets against the *esx-5*-specific PE/PPE epitopes were detected in the *Mtb* Δ*ppe25-pe19*-immunized mice, in contrast to the responses found in the WT *Mtb*-immunized mice (S4 Fig), which displayed characteristics that were similar between *esx-5*-associated and non-*esx-5*-associated (Fig 2B) epitopes. In the *Mtb* Δ*ppe25-pe19*- or *Mtb* WT-immunized groups, we detected comparable frequencies of CCR6^+^ CXCR3^+^ or CD27^-^ PD-1^+^ cells in the TNF-α^+^ and IFN-γ^+^ single positive, TNF-α^+^ IFN-γ^+^ double positive and IL-2^+^ TNF-α^+^ IFN-γ^+^ triple positive functional Th1 subsets, specific to the representative PPE25:1–20 shared epitope (Fig 3A and 3B). These results showed that the differentiation status of the functional Th1 subsets were very similar subsequent to immunization with the *Mtb* WT- or *Mtb* Δ*ppe25-pe19*.

Therefore, compared to the WT *Mtb*, the *Mtb* Δ*ppe25-pe19* strain induces a similar range of differentiated cross-reactive Th1 effectors specific to the shared PE/PPE epitopes, which also recognize the ESX-5-associated PE/PPE virulence-related factors, with very slight differences in the proportions of TNF-α^+^ or IFN-γ^+^ single positive cells.

### ESX-1-linked immunogenic properties of the *Mtb* Δ*ppe25-pe19* strain

**Fig 4:**
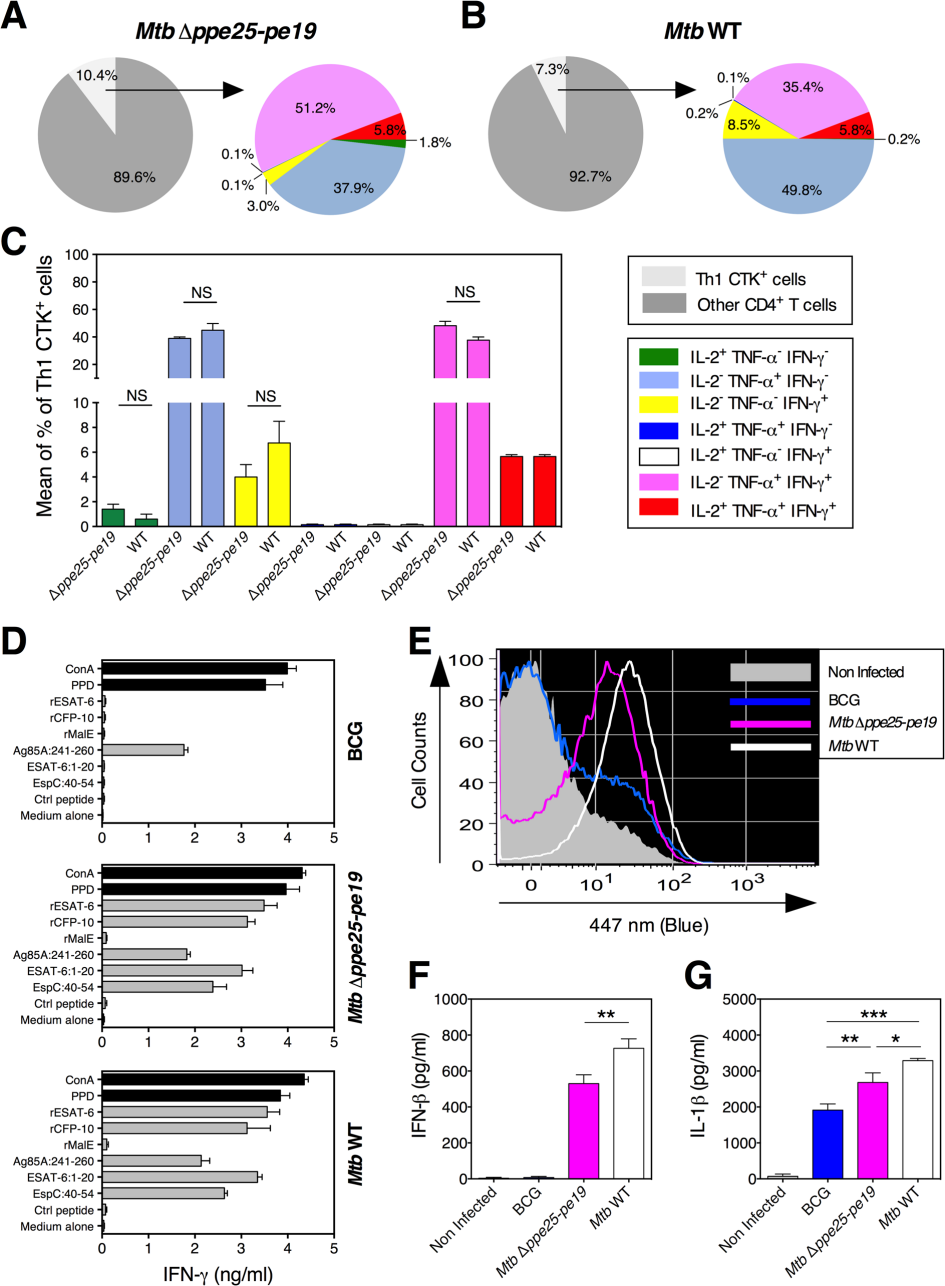
Immunogenic features of the *Mtb* Δ*ppe25-pe19* strain linked to its functional ESX-1 secretion system. Frequencies of different Th1 cytokine-producing splenic CD4^+^ T effectors, at 4 weeks p.i., in C57BL/6 mice (*n* = 5 per group) immunized the *Mtb* Δ*ppe25-pe19* (A) or the *Mtb* WT strain (B) and stimulated *in vitro* with 10 μg/ml of the ESAT-6:1–20 peptide. C) Means ± SD of the frequencies of each Th1 subset, compared between the *Mtb* Δ*ppe25-pe19*- and the *Mtb* WT-immunized mice. NS = statistically not significant as determined by Mann-Whitney test. The immunized mice were those studied for PE/PPE-specific responses in the Fig 2. D) C57BL/6 mice (*n* = 2 per group) were immunized s.c. with 1 x 10^6^ CFU/mouse of BCG, *Mtb* Δ*ppe25-pe19* or *Mtb* WT strain. At 4 weeks p.i., IFN-γ T-cell responses were studied against ESAT-6, CFP-10 and EspC ESX-1 antigens. EspC:40–54 is an immunodominant I-A^b^-restricted epitope that we recently identified by epitope mapping. E) Phagosomal rupture induced in differentiated THP-1 macrophages, infected at MOI of 1, with the *Mtb* Δ*ppe25-pe19* strain as compared to the *Mtb* WT and to BCG Pasteur, as determined at day 3 post infection by the CCF-4-based FRET inhibition assay. In parallel, IFN-β (F) and IL-1β (G) were quantified in the culture supernatants of these infected THP-1 cells at 24 h post infection. *, **, *** = statistically significant, as determined by One Way ANOVA test with Tukey’s correction for multiple comparisons, *p*<0.05, *p*<0.005 or *p*<0.001, respectively.

One of the most relevant properties of the *Mtb* Δ*ppe25-pe19* candidate vaccine is its capacity to secrete ESX-1 virulence determinants ESAT-6 and CFP-10 [40], while displaying a strongly attenuated phenotype relative to parental H37Rv *Mtb* [5, 10]. Virulence comparison test in SCID mice showed that *Mtb* Δ*ppe25-pe19* was slightly more virulent compared to BCG Danish (S5 Fig). The *Mtb* Δ*ppe25-pe19* attenuation profile resembles that of BCG strains belonging to the DU2 IV group (BCG Phipps, BCG Frappier, BCG Pasteur, BCG Tice), which also showed somewhat elevated virulence relative to BCG Danish in a recent comparative study of 13 BCG strains [41, 42]. We further characterized the fine-tuned Th1 immunity specific to ESAT-6 in *Mtb* Δ*ppe25-pe19*- or WT *Mtb*-immunized mice. Compared to the PE/PPE-specific responses, the levels of ESAT-6-specific Th1 cytokine released by splenocytes were generally stronger in both groups (S2 Fig). The distribution of ESAT-6-specific Th1 subsets (Fig 4A–4C) was distinct from that of PE/PPE-specific Th1 subsets (Fig 2A–2C). Comparatively, the ESAT-6-specific response was characterized by decreased percentages of IL-2^-^ TNF-α^+^ IFN-γ^-^ (blue) single positive cells for the benefit of IL-2^-^ TNF-α^+^ IFN-γ^+^ (purple) double positive cells. This suggests that the distribution of Th1 subsets can vary as a function of the antigen specificity following vaccination, probably linked—among others—to the different level of expression and secretion patterns of these different antigens. In addition to ESAT-6- and CFP-10-specific responses, Δ*ppe25-pe19*-immunized mice mounted strong T-cell responses against EspC (Rv3615c), another ESX-1 substrate (Fig 4D), which is also considered as a protective immunogen [43].

In addition to Th1 cells specific to ESX-1/ESX-5-related antigens, other properties of the *Mtb* Δ*ppe25-pe19* strain, including ESX-1-mediated triggering of innate immunity [29–32, 44, 45], may also take part in the improved protective capacity of this strain. A major characteristic feature of ESX-1-proficient mycobacteria is their capacity to induce phagosomal rupture in infected host cells [46, 47], which is followed by the activation of numerous pathways of innate immune responses. These include the cytosolic translocation of mycobacterial DNA, detected by the host cyclic GMP-AMP synthase (cGas) and activation of the STING/TBK/IRF3 pathway. This process leads to the production of IFN-β, as well as the activation of Absent In Melanoma 2 (AIM2) inflammasome/caspase-1 pathway, which contributes to the release of active IL-1β [29–32, 44, 45] and IL-18-mediated noncognate IFN-γ production [48]. Using a FRET method, based on the accessibility of the intrinsic β-lactamase activity of the phagocytosed mycobacteria to the host cytosol [46, 47], we demonstrated that the *Mtb* Δ*ppe25-pe19* strain, in contrast to BCG, is able to induce such phagosomal rupture (Fig 4E). Unlike BCG, the *Mtb* Δ*ppe25-pe19* strain induced secretion of IFN-β by the infected macrophages, albeit at a lesser extent than the virulent *Mtb* WT strain (Fig 4F). Similarly, the *Mtb* Δ*ppe25-pe19* strain induced significantly more IL-1β release than the BCG strain (Fig 4G). These important properties may also take part in the previously reported improved protective capacity of the *Mtb* Δ*ppe25-pe19* strain in comparison to BCG [5].

### Induction of PE/PPE-specific Th1 cells by peptide immunization

**Fig 5:**
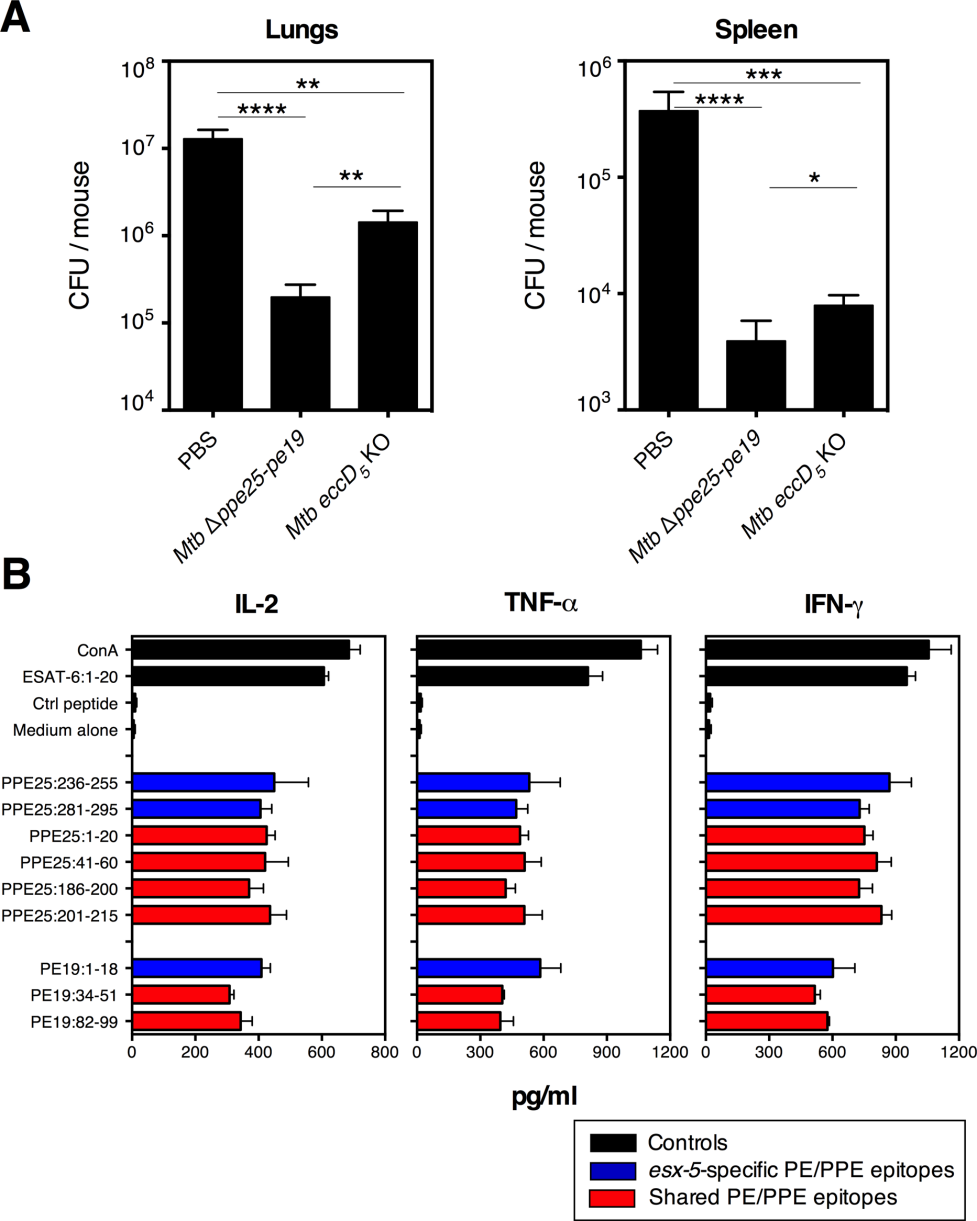
Comparative protective effects of *Mtb* Δ*ppe25-pe19* or *Mtb eccD*_*5*_ KO strain and induction of ESX-5-related PE/PPE specific T-cell responses by immunization with synthetic peptides. A) C57BL/6 mice (*n* = 6 per group), left unvaccinated or immunized s.c. with 1 x 10^6^ CFU/mouse of *Mtb* Δ*ppe25-pe19* or *Mtb eccD*_*5*_ KO strain, were challenged at 4 weeks p.i. via aerosol route with ≈ 200 CFU/mouse of *Mtb* H37Rv WT strain. One month post-challenge, the mycobacterial loads were determined in the lungs and spleen of individual mice. NS = not significant, *, **, ***, **** = statistically significant, as determined by One Way ANOVA test with Tukey’s correction for multiple comparisons, *p*<0.05, *p*<0.005, *p*<0.001 or *p*<0.0001, respectively. B) IL-2, TNF-α and IFN-γ production, detected in the culture supernatants of splenocytes from mice (*n* = 3 per group), immunized with individual synthetic PE/PPE peptides formulated in CpG(DOTAP) at 10 days p.i. Error bars represent ± SD. The results are representative of two independent experiments.

By comparative immunological investigation of the *Mtb* Δ*ppe25-pe19* and *Mtb eccD*_*5*_ KO strains, we previously showed that the former induces robust cross-reactive CD4^+^ T cells against ESX-5-associated PE/PPE and also against a plethora of other PE/PPE antigens, while the latter, which is largely deficient in PE/PPE export/secretion, induces no T-cell response to the panel of PE/PPE epitopes that we selected (S1 Table) [5]. Consistent with our previous observations [5], the *Mtb* Δ*ppe25-pe19* strain displayed a better protective potential than the *Mtb eccD*_*5*_ KO strain (Fig 5A). Based on this observation, we hypothesized that Th1 immunity to PE/PPE antigens may contribute to the cellular mechanisms of TB protection. To experimentally test this hypothesis and to directly evaluate the contribution of PE/PPE-specific Th1 cells in the protection, we established an immunization protocol to induce PE/PPE-specific Th1 responses, not with the live attenuated *Mtb* Δ*ppe25-pe19* vaccine, with complex multifaceted immunological properties (Fig 4), but by use of PE/PPE-derived synthetic peptides (S1 Table).

**Fig 6:**
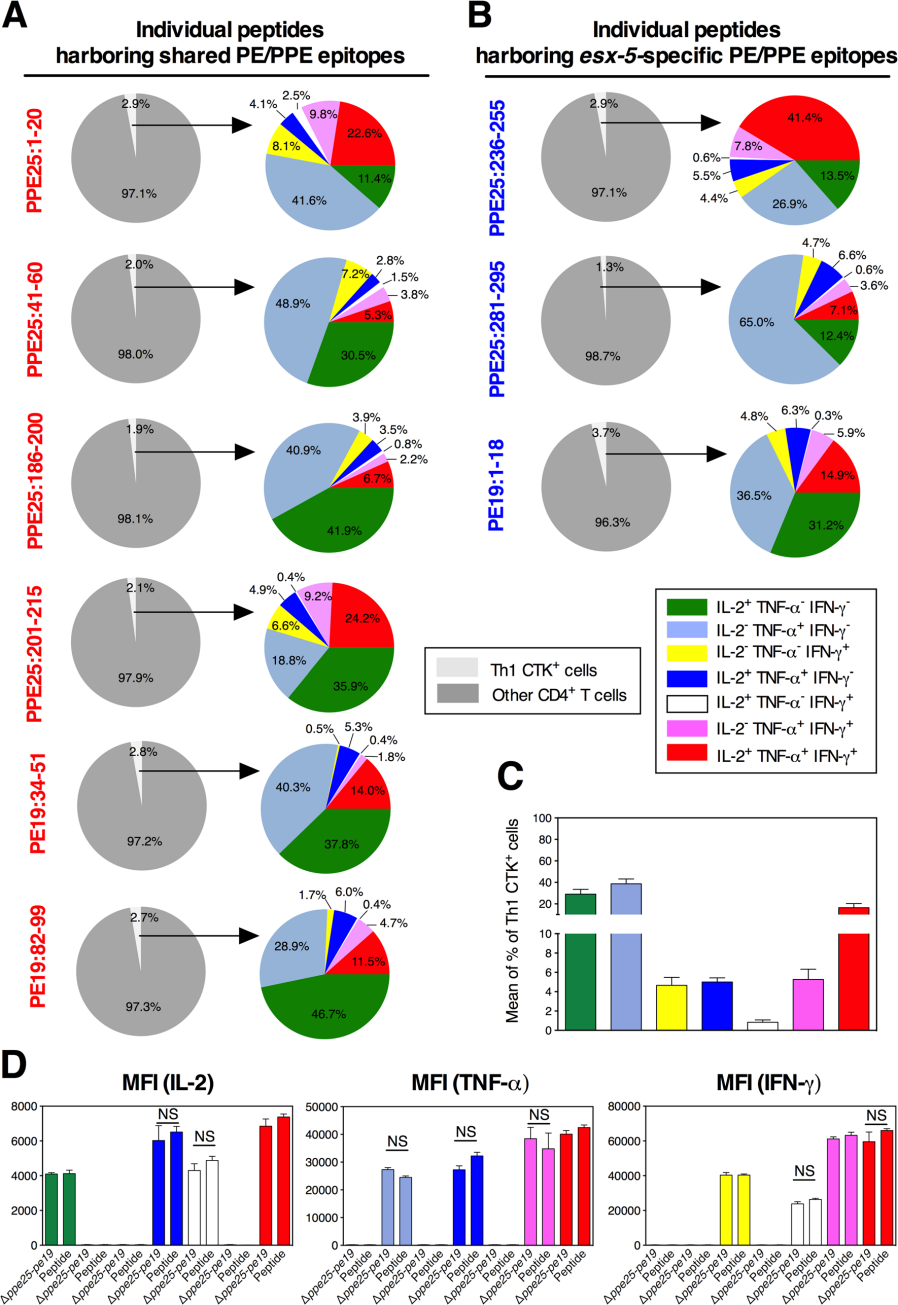
Th1 effector subsets specific to PE/PPE epitopes subsequent to vaccination with individual synthetic peptides. Percentage and composition of CD4^+^ T splenic effectors of C57BL/6 mice (*n* = 3 per group) vaccinated with individual synthetic peptides containing PE19- and PPE25-derived epitopes, either highly specific to *esx-5* (A) or shared by other PE/PPE proteins (B) coded outside *esx-5* region. C) Means ± SD of the frequencies of each Th1 subset, as cumulated for all the studied peptides, in the immunized groups. (D) The geometric Mean Fluorescence Intensities (MFI) of intracellular IL-2, TNF-α or IFN-γ in each of the antigen-specific functional Th1 subsets, as determined in the spleen of mice immunized with *Mtb* Δ*ppe25-pe19* or with PPE25:1–20, as a representative peptide. The results are representative of two independent experiments.

C57BL/6 mice (*n* = 3 per group) were immunized s.c. twice at a 10-day interval with each of the individual PE/PPE-derived peptides. As adjuvant TLR9 agonist CpG oligodeoxynucleotide, associated with the liposomal transfection reagent DOTAP (N-[1-(2,3-DioleOyloxy)]-N,N,N-TrimethylAmmonium Propane methylsulfate) was used. At day 10 after the second injection, antigen-specific production of IL-2, TNF-α, and IFN-γ by CD4^+^ T splenocytes was readily detected (Fig 5B). However, for all of the epitopes, the amounts of these cytokines produced by the splenocytes of the peptide-immunized mice were weaker than the levels produced by their mycobacteria-immunized counterparts (S2 Fig). Results from an ICS assay performed in mice immunized with each peptide (Fig 6A–6C) showed increased frequencies of IL-2^+^ TNF-α^-^ IFN-γ^-^ (green) single positive, IL-2^+^ TNF-α^+^ IFN-γ^-^ (dark blue) double positive and IL-2^+^ TNF-α^+^ IFN-γ^+^ (red) triple positive Th1 cells for each epitope, compared to the frequencies observed in their *Mtb Δppe25-pe19*-immunized counterparts (Fig 2C). Moreover, the terminally differentiated TNF-α^+^ single positive cells constituted the major Th1 cell subset in these peptide-immunized mice (Fig 6A–6C). The PE/PPE-specific Th1 cells induced by peptide or *Mtb* Δ*ppe25-pe19* immunization notably displayed the following functional and phenotypic features: (i) Fluorescence Intensities (MFI) of the ICS staining for each Th1 cytokine, which are proportional to the amounts of cytokine production per cell (Fig 6D), and (ii) the differentiation status of the TNF-α^+^ and IFN-γ^+^ single positive, TNF-α^+^ IFN-γ^+^ double positive and IL-2^+^ TNF-α^+^ IFN-γ^+^ triple positive functional Th1 subsets, in terms of CCR6, CXCR3, CD27 and PD-1 surface expression (S6 Fig and Fig 3).

### Contribution of PE/PPE-specific Th1 responses to anti-mycobacterial protection

**Fig 7:**
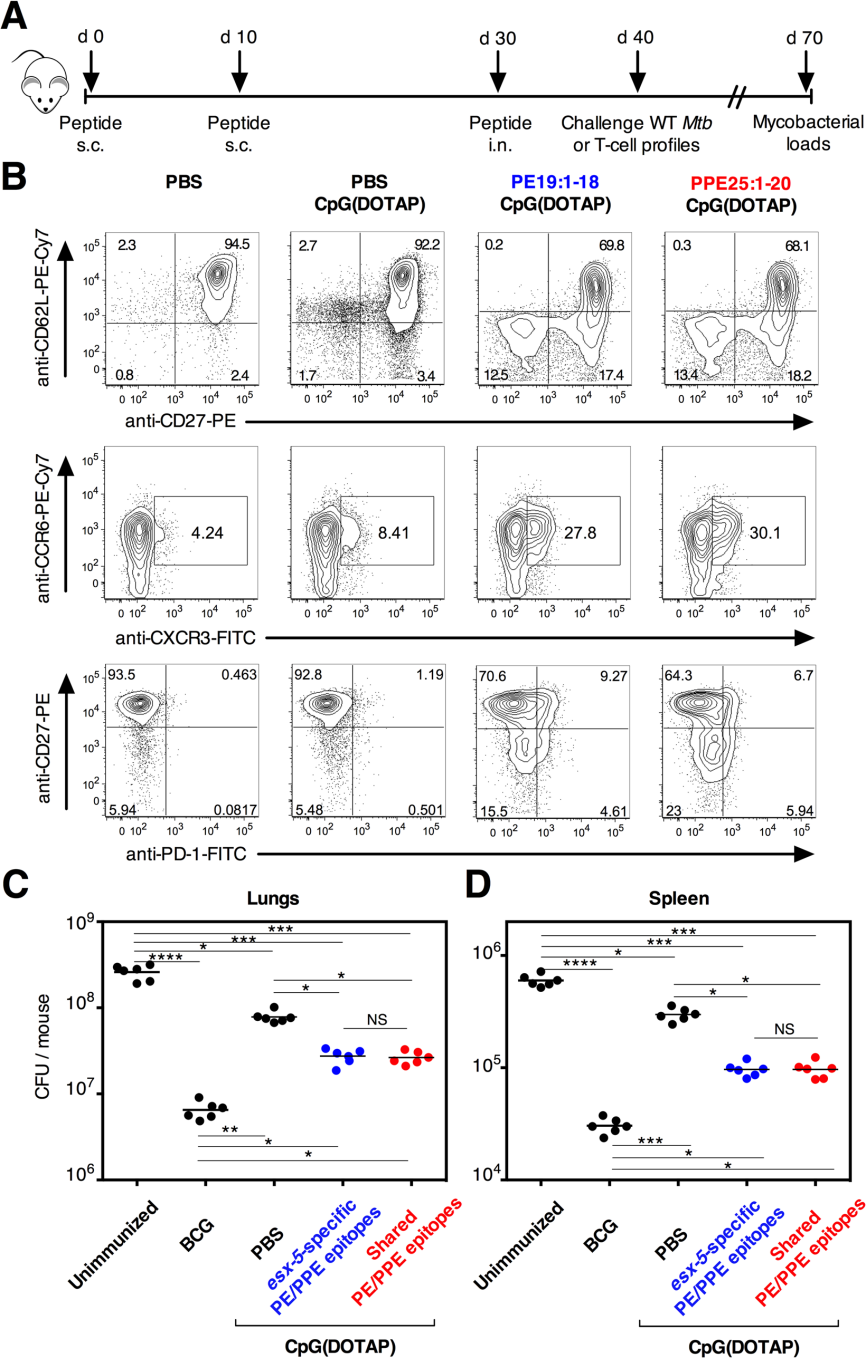
Evaluation of the protective effect of PE/PPE immunization against *Mtb* infection. A) Immunization regimen with PE/PPE-derived peptides. C57BL/6 mice (*n* = 6 per group) were injected with PBS or vaccinated twice s.c. at 10-days interval with individual or mixtures of selected PE/PPE-derived peptides, formulated in CpG(DOTAP). Peptide-immunized mice were boosted with the same individual or mixted PE/PPE peptides, formulated in CpG(DOTAP), via i.n. route at day 30. B) Expression of T-cell activation/migration/differentiation markers by the lung CD3^+^ CD4^+^ T cells in PE/PPE-immunized or the control mice, as studied *ex vivo* at day 40. See furthermore S8A Fig. C-D) Protective potential of such immunization against an aerosol challenge with virulent *Mtb*. Mice were immunized according to (A) with PE/PPE peptide mixtures of (i) *esx-5*-specific epitopes (PE19:1–18, PPE25:281–295 and PPE25:236–255 peptides), or (ii) shared epitopes (PE19:34–51, PPE25:1–20 and PPE25:201–215 peptides). They were then challenged at day 40 via aerosol route with ≈ 200 CFU/mouse of *Mtb* H37Rv WT strain. One month post-challenge, the mycobacterial loads were determined in the lungs (C) and spleen (D) of individual mice. NS = not significant, *, **, ***, **** = statistically significant, as determined by One Way ANOVA test with Tukey’s correction for multiple comparisons, *p*<0.05, *p*<0.005, *p*<0.001 or *p*<0.0001, respectively.

We further evaluated directly the contribution of the anti-PE/PPE poly-specific Th1 cells, systematically and locally induced by use of the PE/PPE-derived synthetic peptides, in the protection against virulent *Mtb*. For a better understanding of the protective adaptive immunity in terms of fine specificity of T cells, we compared the protective potential of Th1 cells either specific to the *esx-5*-associated PE/PPE epitopes or to the shared epitopes. C57BL/6 (*n* = 6 per group) mice were vaccinated according to the protocol schematized in the Fig 7A with individual PE/PPE peptides (for prior immune response study) or mixtures of such peptides (for protection studies). PE/PPE peptides which harbor *esx-5*-specific or shared epitopes were formulated in CpG(DOTAP). Moreover, since previous works demonstrated that mucosal local immunity and the homing of effector T cells from the lung vasculature to the parenchyma is crucial for the pulmonary TB protection [49–52], the mice were also boosted i.n. with homologous PE/PPE peptides 10 days before the challenge. In the pulmonary CD4^+^ T-cell compartment of these mice, we detected in *ex vivo* tests increased percentages of CD27^-^ CD62L^-^, CCR6^+^ CXCR3^+^, CD27^-^ PD-1^+^ (Fig 7B), as well as CD44^hi^ (S7A Fig) cells, representing a hallmark of migratory antigen-specific poly-functional effector T cells of the peripheral tissues.

The protective potential of immunization with the peptide mixtures was compared to that of vaccination with BCG 1173P2 Pasteur strain. At day 40, vaccinated mice or untreated controls were aerosol infected with the virulent *Mtb* H37Rv strain, delivered at dose of ≈ 200 CFU/lungs. At day 70, determination of the mycobacterial loads in the lungs (Fig 7C) and spleen (Fig 7D) showed that immunization with the mixtures of PE/PPE peptides, either specific to the *esx-5* region or shared with other homologs, induced a significant protection, which was only partially due to the effect of the adjuvant alone.

**Fig 8:**
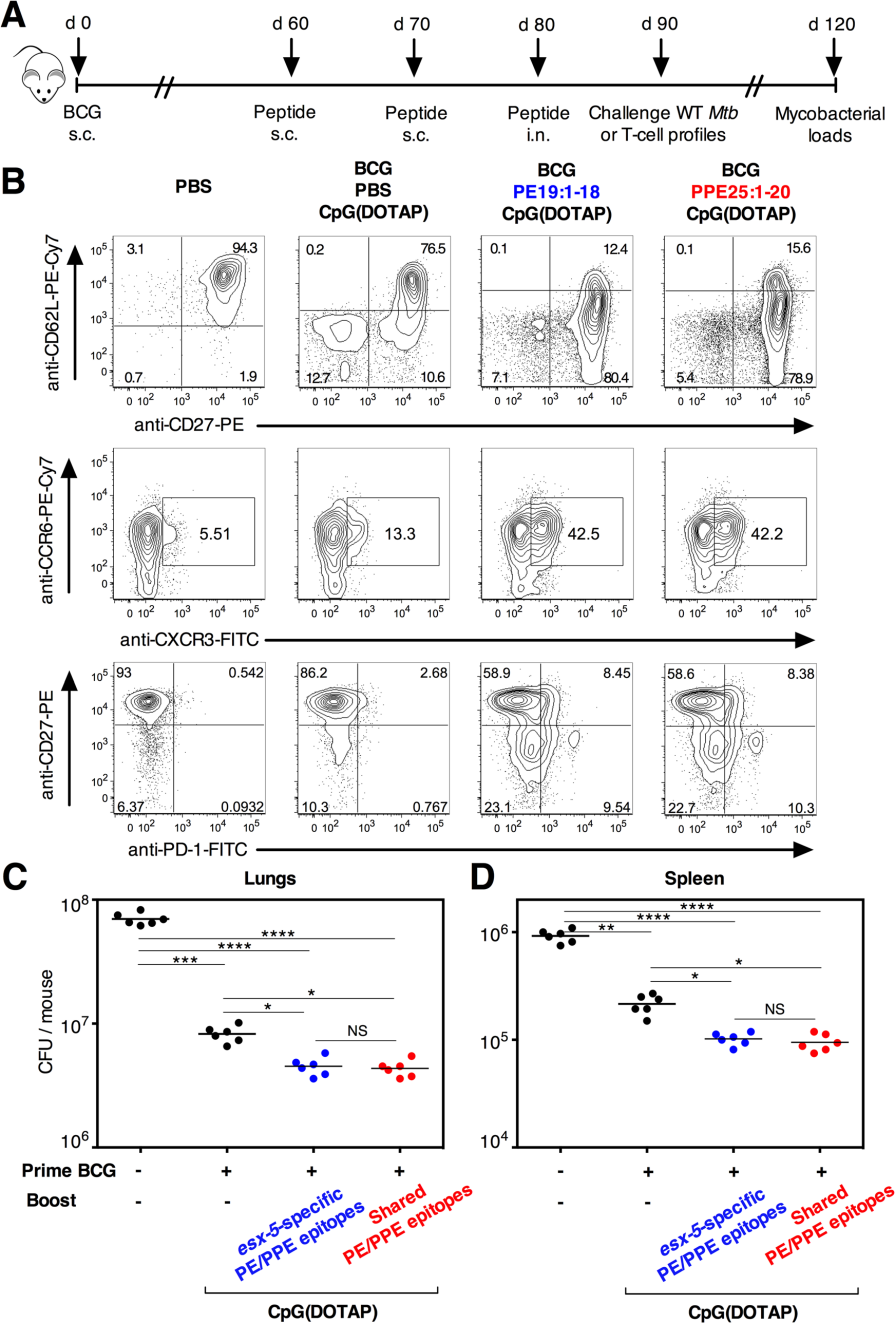
Improved protection with PE/PPE boosting in BCG-primed mice. A) BCG priming and boosting protocol with PE/PPE-derived peptides. C57BL/6 mice (*n* = 6 per group) were injected with PBS or immunized s.c. with BCG. Two months later, mice were vaccinated twice s.c. at 10-days interval with individual or mixtures of PE/PPE-derived peptides formulated in CpG(DOTAP), followed by an i.n. administration of the same peptides. B) Expression of T-cell activation/migration/differentiation markers by the lung CD3^+^ CD4^+^ T cells of BCG-primed, PE/PPE-boosted mice at day 90. See furthermore S8B and S8C Fig. Mice were challenged 10 days after the last immunization (day 90) with ≈ 200 CFU/mouse of *Mtb* H37Rv WT strain via aerosol, as determined by day 1 post challenge by CFU counting in the lungs. One month post challenge, the mycobacterial loads were determined in the lungs (C) and spleen (D) of individual mice. NS = not significant, *, **, ***, **** = statistically significant, as determined by One Way ANOVA test with Tukey’s correction for multiple comparisons, *p*<0.05, *p*<0.005, *p*<0.001 or *p*<0.0001, respectively.

BCG has an intact ESX-5 secretion system and induces T-cell immunity against all the selected PE/PPE epitopes (S7 Fig). We thus evaluated the effect of BCG priming and PE/PPE boosting on immune responses and TB protection following immunization with PE/PPE epitopes, as detailed in the Fig 8A In the lungs of these mice, as determined *ex vivo*, we observed increased percentages of CD27^-^ CD62L^-^, CCR6^+^ CXCR3^+^, CD27^-^ PD-1^+^ (Fig 8B), and CD44^hi^ (S7B Fig) cells, within the CD4^+^ T-cell compartment, as well as increased total numbers of CD4^+^ T cells (S8C Fig). BCG priming followed by PE/PPE boosting significantly improved the control of mycobacterial growth in the lungs (Fig 8C) and limited the mycobacterial dissemination to the spleen (Fig 8D).

Altogether, our results highlight the protective capacity of these PE/PPE proteins as immunogens and unravel part of the immune mechanisms of the remarkable protective property of the *Mtb* Δ*ppe25-pe19* vaccine candidate.

## Discussion

In the present study, we demonstrated that PE/PPE-specific Th1 cells contribute to the cellular protective immune mechanisms developed by the live attenuated *Mtb* Δ*ppe25-pe19* TB vaccine candidate that we recently generated [5, 10]. Secretion or export to the bacterial cell-envelop is a prerequisite for most mycobacterial antigens to access the antigen presentation machinery inside the host innate immune cells and for specific detection by effector CD4^+^ T cells [3]. A large number of PE/PPE proteins are exported/secreted via the ESX-5 T7SS [24], although a few PE/PPE proteins might also be handled via the SecA general secretory pathway [53]. The biological activities of PE/PPE proteins are thus likely linked to their cell surface-associated or extracellular localization, which may also explain their notable immunogenicity [14, 54–58]. It has been proposed that the duplication and random insertion of the *pe/ppe* genes throughout the *Mtb* genome may have led to their transcriptional control by a random assortment of unrelated promoters and regulators, which could result in substantial degrees of variability in their expression profiles during different phases of infection [59]. Besides, there exist compelling sequence homologies among the PE/PPE members resulting from gene duplication. This context may lead to the consecutive availability of groups of PE/PPE epitopes during various phases of infection, despite the variability in the expression profiles of the PE/PPE members from which they derive. Such properties may contribute to the interest of these proteins in the protective immunity against chronic mycobacterial infections.

The attenuated *Mtb* Δ*ppe25-pe19* strain is deficient only in five PE/PPE proteins, i.e., PPE25-27 and PE18-19, which are coded inside the *esx-5* region [5, 10]. However, due to the intact secretion machinery of the ESX-5 system, the *Mtb* Δ*ppe25-pe19* strain has preserved the capacity to export/secrete substantial numbers of other PE/PPE proteins encoded elsewhere in the *Mtb* genome (S1B Fig, right) [24]. Immunization with the *Mtb* Δ*ppe25-pe19* strain thus induces T-cell immunity against these PE/PPE proteins, including the non-*esx-5*-associated members, which display compelling sequence homologies with the missing *esx-5*-coded PE/PPE antigens [5]. Therefore, the antigenic repertoire of the *Mtb* Δ*ppe25-pe19* strain remains virtually comparable to that of the WT *Mtb* strain despite its strongly attenuated virulence phenotype.

Immune correlates of TB protection remain elusive. So far, in human, there is no consensus whether the induction of poly-functional Th1 cells and the distribution of the various Th1 subsets are markers of either active TB disease or of protective immunity in latent TB infection [60–62]. Single positive IL-2^+^ Th1 cells are usually central memory T cells, able to proliferate and differentiate to effector memory and/or effector cells, while single positive IFN-γ^+^ or TNF-α^+^ Th1 cells are terminally differentiated, not proliferative and short-lived populations [28]. The accumulation of TNF-α^+^ single positive cells is considered as predictor of diagnosis of active TB [63]. Consistently, it is admitted that in chronic diseases like TB, the continuous antigenic stimulation of T cells leads to the loss of both memory potential and poly-functionality, which results in terminally differentiated T cells that only produced IFN-γ or TNF-α. In mice, poly-functional IL-2^+^ TNF-α^+^ IFN-γ^+^ Th1 responses against prominent mycobacterial immunogens cells display a positive correlation with proliferative capacity, indicative of their effector capacity. In the mouse model, these cells are considered the most reliable parameter able to control the growth and dissemination of *Mtb in vivo* [27]. Here, we showed that immunization of mice with the *Mtb* Δ*ppe25-pe19* strain induces marked IL-2^-^ TNF-α^+^ IFN-γ^+^ double positive and IL-2^+^ TNF-α^+^ IFN-γ^+^ triple positive poly-functional Th1 effector cells specific to a panel of PE/PPE epitopes. Notably, most PE/PPE-specific triple positive Th1 splencoytes in the *Mtb* Δ*ppe25-pe19*-immunized mice exhibited a CXCR3^+^ CCR6^+^ PD-1^+^ phenotype, as a hallmark of effector memory and protective T-cell population. [35–38]. Expression of the PD-1 inhibitory receptor by Th1 cells has been recently shown to be of utmost important in the TB protection via the negative regulation of IFN-γ-over-expressing CD4^+^ T cells [38]. We further compared, in mice immunized with the *Mtb* WT or the *Mtb* Δ*ppe25-pe19* strain, the profiles of functional Th1 subsets specific to PE/PPE epitopes, which are either highly specific to the *esx-5* region or shared with PE/PPE homologs coded outside of *esx-5* [5]. As expected, the *Mtb* Δ*ppe25-pe19* strain does not induce T-cell responses against the first group of epitopes. However, the responses were comparable in the *Mtb* Δ*ppe25-pe19*- or *Mtb* WT-immunized groups against the shared PE/PPE epitopes in terms of their fine composition of Th1 effector subsets and their differentiation status. Therefore, despite its attenuation, the *Mtb* Δ*ppe25-pe19* strain generates bi- and poly-functional Th1 cells, which recognize the PE/PPE antigens that it lost, with the diverse Th1 subsets distributed comparably to *Mtb* WT.

We further demonstrated that PE/PPE-specific Th1 responses contribute actively to the anti-TB immunity. This is shown by the induced Th1 cells, as well as the recruitment and activation of effector T cells in the lungs, following systemic and local immunization of mice with selected PE/PPE epitopes, formulated in CpG(DOTAP) adjuvant. Importantly, the mycobacterial PE/PPE epitopes, either *esx-5*-sepcific, with no homologies with other PE/PPE proteins, or shared with PE/PPE homologs coded outside of *esx-5*, induce similar levels of protection. Therefore, it can be proposed that the shared surrogate PE/PPE homologs in the *Mtb* Δ*ppe25-pe19* strain compensate for the loss of the *esx5*-specific epitopes. In addition, booster immunization with such PE/PPE epitopes in BCG-primed individuals significantly improved the protection. These results thus show that such PE/PPE proteins represent potent immunogens to be included in TB subunit vaccines or as boosters.

Despite its attenuated phenotype, the *Mtb* Δ*ppe25-pe19* strain remains able to induce Th1 responses against ESX-1-associated virulence factors, including at least ESAT-6, CFP-10 and EspC, known as protective immunogens [40, 43, 64–69]. Moreover, the presence of a functional ESX-1 system preserves the capacity of the *Mtb* Δ*ppe25-pe19* strain at inducing ruptures in the phagosomal membrane inside the host phagocytes. The phagosomal rupture results in a phagosome-cytosol communication, leading to the release of mycobacterial compounds, including the extracellular mycobacterial DNA, to the host cytosol. Mycobacterial DNA is then sensed by cGas and ultimately activates IFN-β gene transcription [29–32]. We showed that *Mtb* Δ*ppe25-pe19*- (but not BCG-) infected macrophages secrete IFN-β. On the other hand, detection of mycobacterial DNA by the cytosolic AIM2 inflammasome increases caspase-1 activation and contributes significantly to the release of mature IL-1β [70]. It has been shown that following the ESX-1-dependent phagosomal rupture, the ESX-5 T7SS, via still unknown mechanisms, activates inflammasome and caspase-1, which results in IL-1β release [71]. It is noticeable that the ESX-5 mutant *Mtb* Δ*ppe25-pe19* strain is only deficient in five PE/PPE *esx-5*-associated proteins, and still harbors a functional ESX-5 system. This property seemingly confers to this strain an increased capacity to activate inflammasome and IL-1β release. Even though the role of the type-I IFN in the induction of protective immune responses remains elusive, that of IL-1β is instrumental in the anti-mycobacterial host defense [72]. Collectively, the immunological fine characterization presented in this study emphasizes the unique properties of *Mtb* Δ*ppe25-pe19* strain to stimulate host immunity in terms of both antigenic repertoire and innate immune responses.

While the safety profile for the *Mtb* Δ*ppe25-pe19* strain in SCID or immune-competent mice [5, 10] is within the range of BCG strains [41, 42], work is in progress to introduce a second attenuating gene deletion in order to satisfy the Geneva Consensus recommendations for novel live TB vaccines [73]. This process shall provide an *Mtb* Δ*ppe25-pe19* derivative with increased security and safety, but unaffected advantageous immunological profile, to be used as a new veterinary or human TB vaccine.

## Materials and Methods

### Mycobacteria

*Mtb* Δ*ppe25-pe19* [10] and *Mtb* WT H37Rv strains were grown in Dubos broth, complemented with Albumine, Dextrose and Catalase (ADC, Difco, Becton Dickinson, Le Pont-de-Claix, France). The bacterial contents were determined by OD measurement at 600 nm. CFU were counted on Middlebrook 7H11 solid Agar medium after 18 days of incubation at 37°C. All experiments with pathogenic mycobacteria were performed in an L3 protection level laboratory, in accordance with the hygiene and security recommendations of Institut Pasteur.

### Peptides

The synthetic peptides which contain MHC-II-restricted antigenic epitopes were synthesized by PolyPeptide Group (Strasbourg, France), reconstituted in H_2_O containing 5% dimethyl sulfoxide (DMSO) (Sigma-Aldrich, France) and stored at -20°C.

### Mice and immunizations

Six-to-eight week-old female C57BL/6 (H-2^b^) mice (Janvier, Le Genest-Saint-Isle, France) were immunized by s.c. injection, at the base of the tail, of 1 x 10^6^ CFU/mouse of *Mtb* Δ*ppe25-pe19* or *Mtb* WT strains in 200 μ1 volume. Immunizations with peptides were performed by two s.c. injections at a 10-day interval, with 100 μg/mouse of individual peptide, formulated with 30 μg of CpG 1826 oligodeoxynucleotides as adjuvant (Sigma-Aldrich, France), 60 μl of liposomal transfection reagent DOTAP (N-[1-(2,3-DioleOyloxy)]-N,N,N-TrimethylAmmonium Propane methylsulfate, Roche, France) and 10 μl of Opti-MEM (Life Technologies, France) in a final volume of 200 μl. The liposomal transfection reagent DOTAP optimizes the adjuvant effect of CpG by conducting it to the endosomal sites where the intracellular TLR-9 receptor is localized.

### Ethics statement

Studies in immunocompetent mice were performed in agreement with guidelines of the European and French guidelines (Directive 86/609/CEE and Decree 87–848 of 19 October 1987), after approval by the Institut Pasteur Safety, Animal Care and Use Committee and under local ethical committee protocol agreement # CETEA 2013–0036 and CETEA 2012–0005. Analysis of virulence in SCID mice was approved by the UK Home Office (HO) regulations for animal experimentation which requires a HO-approved licence and approval from local ethical committees of Public Health England, Porton Down (Licence number PPL30/2704) and London School of Hygiene and Tropical Medicine (LSHTM) Animal Welfare and Ethical Review Board (Authorization # 70/6934).

### T-cell cytokine secretion assay

Splenocytes from immunized mice were cultured in flat-bottom 96-well plates (TPP, Denmark) at 1 x 10^6^ cells per well in HL-1 medium (Biowhittaker, Lonza, France), complemented with 2 mM GlutaMax (Invitrogen, Life Technologies, France), 5 x 10^−5^ M β-mercaptoethanol, 100 U/ml penicillin and 100 μg/ml streptomycin (Sigma-Aldrich, France) in the presence of 10 μg/ml of individual peptides. After 12, 48 and 72 hours of incubation at 37°C and 5% CO_2_, IL-2, TNF-α and IFN-γ were respectively quantified in the culture supernatants by ELISA as previously described [8]. Monoclonal antibodies (mAbs) specific to IL-2 (clone JES6-1A12 for coating and clone JES6-5H4 for detection) or IFN-γ (clone AN-18 for coating and clone R4-6A2 for detection) were from BD Pharmingen (Le pont-de-Claix, France). Anti-TNF-α mAbs (clone 1F3F3D4 for coating and clone XT3/XT22 for detection) were from eBioscience.

### Cytometry

Single-cell suspensions from spleen of immunized mice were obtained by tissue dissociation, homogenization and passage through 100 μm-pore filter. Cells were cultured at 7.5 x 10^6^ cells/well in the presence of 1 μg/ml anti-CD28 (clone 37.51) and 1 μg/ml of anti-CD49d (clone 9C10-MFR4.B) mAbs (BD Pharmingen) together with 1 x 10^6^ cells/well syngenic bone-marrow dendritic cells, loaded with 10 μg/ml of homologous or control peptide during 1h, followed by 5h of incubation with Golgi Plug (BD Pharmingen), according to manufacture’s instructions. Cells were then harvested, washed twice with PBS containing 3% Fetal Bovine Serum (Invitrogen, Life Technologies, France) and 0.1% NaN_3_ (FACS buffer) and incubated for 15 minutes at 4°C with FcγII/III receptor blocking anti-CD16/CD32 (clone 2.4G2) mAb. Cells were then incubated for 25 minutes with appropriate dilutions of AlloPhycoCyanin (APC)-eFluor780-anti-CD3ε and PB-anti-CD4 mAbs (BD Pharmingen) at 4°C and sheltered from light. The stained cells were washed twice in FACS buffer, permeabilized by use of Cytofix/Cytoperm kit (BD Pharmingen). Cells were then washed twice with PermWash 1X buffer from the Cytofix/Cytoperm kit and incubated with appropriate dilutions of PerCP-Cyanine_5.5_-anti-IL-2 (clone JES6-5H4, eBioscience), PE-anti-TNF-α (clone 554419, BD Pharmingen), and Alexa Fluor_647_-anti-IFN-γ (clone XMG1.2, eBioscience) mAbs during 30 minutes at 4°C. Appropriate staining with control Ig isotypes was performed in parallel. Cells were subsequently washed twice in PermWash buffer, once in FACS buffer and then fixed with 4% paraformaldehyde overnight at 4°C. When indicated, cells were stained at the surface, either *ex vivo* or after *in vitro* simulation before ICS, with a cocktail of (APC)-eFluor780-anti-CD3ε, PB-anti-CD4, PE-Cy7-anti-CCR6 (Sony Biotechnology), FITC-anti-CXCR3 (eBioscience), FITC-anti-PD-1 (Biolegend) and PE-Cy7-anti-CD27 (BD Pharmingen) mAbs. We preliminarily checked that the expression of CCR6, CXCR3, CD27 and PD-1 markers did not change during the short *in vitro* stimulation required for ICS.

To study the phenotype of the pulmonary T cells, lungs were first disaggregated by treatment with 400 U/ml type IV collagenase and DNase I (Roche). Following a 45-min incubation at 37°C, single-cell suspensions were prepared by use of GentleMacs (Miltenyi) and passage through 100-μm nylon filters (Cell Strainer; BD Falcon). Cell suspensions were then enriched in lymphocytes by 20-min centrifugation at 3000 rpm at RT on Ficoll gradient medium (Lympholyte M, Cedarlane Laboratories). The cells were then washed twice and stained with a cocktail of (APC)-eFluor780-anti-CD3ε, PB-anti-CD4, PE-anti-CD27, PE-Cy7-anti-CD62L, PE-anti-CD44 (eBioscience) and FITC-anti-CD45RB (eBioscience) mAbs in the presence of FcγII/III receptor blocking mAb. The stained cells were then fixed with 4% paraformaldehyde. The cells were acquired in an LSR Fortessa flow cytometer system by use of BD FACSDiva software (BD Bioscience). Data were analyzed using FlowJo software (Treestar, OR, USA).

### Phagosomal rupture assay and innate immune response analysis

PMA-differentiated THP-1 cells were infected with *Mtb* WT or *Mtb* Δ*ppe-25-pe19* strains at MOI of 1. At day 3 p.i., the phagosomal rupture was assessed by Fluorescence Resonance Energy Transfer (FRET) assay as previously described [47]. Briefly, cells were stained with 8 μM CCF-4 (Cephalosporin core linking a 7-hydroxyCoumarin to a Fluorescein) (Invitrogen) in EM buffer (120 mM NaCl, 7 mM KCl, 1.8 mM, CaCl2, 0.8 mM MgCl2, 5 mM glucose and 25 mM Hepes, pH 7.3) complemented with 2.5 μM probenecid, during 1h at room temperature. Cells were then washed in PBS and stained with APC-anti-CD11b (BD Pharmingen) mAb in FACS buffer. Cells were then fixed with 4% paraformaldehyde overnight at 4°C and were analyzed in a CyAn cytometer (Beckman Coulter, France). Human IL-1β and IFN-β were quantified in the culture supernatants of these infected THP-1 cells at 24h p.i. by use of (DY201-05, R&D Systems) and (41410, PBL Assay Science) kits, respectively.

### Protection assays

Six-to-eigth week-old female C57BL/6 mice were left untreated or were immunized s.c. with 1 x 10^5^ CFU/mouse of BCG (1173P2 Pasteur strain) at day 0 or immunized s.c. twice at days 10 and 20, with 50 μl of each PE/PPE-derived peptide of interest, 30 μg of CpG, 60 μl of DOTAP and 10 μl Opti-MEM contained in 200 μl/mouse. At day 30, peptide-immunized mice received via intra-nasal route under anesthesia 20 μg of each PE/PPE-derived peptide of interest, 20 μg of CpG, 10 μl of DOTAP and 3 μl Opti-MEM contained in 20 μl/mouse. For anesthesia, mice received i.p. 100 μl/mouse of suspension containing weight-adapted quantities of Imalgène_1000_ (Kétamine, i.e., 100 mg/kg, Merial, France) and Rompun 2% (Xylazine solution, 10 mg/kg, Bayer, Germany), prepared in physiological solution. Mice were challenged 10 days after the last immunization by use a homemade nebulizer via aerosol. Five ml of a suspension containing 2.5 x 10^6^ CFU/ml of *Mtb* H37Rv WT strain were aerosolized to reach an inhaled dose of ≈ 200 CFU/mouse, as determined by day 1 p.i. CFU count in the lungs of the challenged mice. The infected mice were placed and manipulated in isolator in A3 protection-level facilities at Institut Pasteur. One month later, the lungs and spleen of the infected mice were individually homogenized by using a MillMixer organ homogenizer (Qiagen, Courtaboeuf, France). Serial 5-fold dilutions were plated on 7H11 Agar medium supplemented with ADC (Difco, Becton Dickinson). The CFU were counted after 18–21 days of incubation at 37°C.

### Statistical analyses

The statistical analyses were performed by use of GraphPad Prism software (GraphPad Software, La Jolla, CA, USA) and Mann-Whitney test for simple comparison or One Way ANOVA test with Tukey’s correction for multiple comparisons in order to determine the statistical significance of obtained data.

### Accession numbers

ID numbers for proteins mentioned in the text according to Tuberculist (http://genolist.pasteur.fr/TubercuList/index.html)

ESAT-6, EsxA: Rv3875

CFP-10, EsxB: Rv3875

EspC: Rv3615c

PE18: Rv1788

PE19: Rv1791

PPE25: Rv1787

PPE26: Rv1789

PPE27: Rv1790

EccD_5_: Rv1795

## Supporting Information

S1 Table Immunogenic regions of ESX-5 associated PE19 and PPE25. PPE25- and PE19-derived peptides, containing MHC-II, I-A^b^-restricted T-cell epitopes, either highly specific to *Mtb esx-5* region (A) or shared by other PE/PPE homologs coded outside this region (B), as previously identified [5].
(TIF)

S1 Fig The *esx-1* and *esx-5* genomic regions of *Mtb* and schematic representation of the ESX-1 and ESX-5 T7SSs. (A) Genetic organization of the *esx-1* and *esx-5* genomic regions of *Mtb*. (B) Schematic representation of ESX-1 and ESX-5 T7SSs which export/secret Esx and PE/PPE proteins. Secretion of ESAT-6 and CFP-10 via ESX-1 is responsible of the induction of specific T cells in immunized mice (B, left). Numerous PE/PPE proteins, either coded inside or outside the *esx-5* region, are exported/secreted via the trans-membrane EccD_5_ channel of the ESX-5 system. Subsequent to immunization with the *Mtb* Δ*ppe25-pe19* strain, secretion of numerous PE/PPE homologs coded outside *esx-5* induces T cells, which via cross reactivity, are able to recognize ESX-5-coded PE/PPE virulence-related factors.
(TIF)

S2 Fig Induction of Th1 cytokine responses specific to *esx-5*-coded PPE25 and PE19 in *Mtb* Δ*ppe25-pe19*- or WT-immunized mice. IL-2, TNF-α and IFN-γ production, as quantified by ELISA in the culture supernatants of splenocytes from C57BL/6 mice (*n* = 5 per group) immunized s.c. with the *Mtb* Δ*ppe25-pe19* or the *Mtb* H37Rv WT strain and stimulated *in vitro* with individual PPE25- and PE19-derived peptides, either highly specific to *esx-5* or shared by other PE/PPE proteins coded outside this region at 4 wks p.i. ESAT-6:1–20 or MalE:100–114 peptides were used respectively as positive or negative controls. Error bars represent SD. The results are representative of two independent experiments.
(TIF)

S3 Fig Results obtained with negative controls in cytometric analyses used to identify functional Th1 subsets in the Fig 1A. Splenocytes from the *Mtb Δppe25-pe19*-immunized C57BL/6 mice shown in the Fig 1 were stimulated *in vitro* with the control MalE:100–114 (A) or homologous peptide (B), prior to surface and intracellular staining with anti-cytokine mAbs (A), or to intracellular staining with control Ig isotypes (B).
(TIF)

S4 Fig Profile of Th1 cytokine-producing CD4^+^ T cells specific to individual *esx-5*-specific PE/PPE epitopes in the *Mtb* WT-immunized mice. (A-B) Th1 cytokine-producing splenic CD4^+^ T effectors of C57BL/6 mice (*n* = 5) at 4 weeks after s.c. injection with 1 x 10^6^ CFU/mouse of *Mtb* WT strain, analyzed as detailed in the legend to the Fig 1. Such Th1 responses against PE/PPE epitopes specific to *esx*-5 were not detected in *Mtb* Δ*ppe25-pe19*-immunized mice.
(TIF)

S5 Fig Virulence of the *Mtb* Δ*ppe25-pe19* strain compared to the BCG Danish vaccine. SCID mice were infected i.v. with 1 x 10^6^ CFU/mouse of the BCG Danish 1331 vaccine or the *Mtb* Δ*ppe25-pe19* strain, as a selected TB vaccine candidate in an independent preclinical virulence trial within the framework of the TBVAC2020 consortium. Control mice received saline only. The weight loss kinetics was followed over a period of 90 days. Animals were euthanized when they reached the human endpoint of >20% weight loss or showed severe clinical signs of disease according to the UK Home Office guidelines referring to the welfare of experimental animals. The *Mtb* Δ*ppe25-pe19* strain displayed only a weakly higher degree of virulence, as compared BCG Danish 1331, which is the most attenuated live TB vaccine [41, 42]. Work is currently in progress to introduce in the *Mtb* Δ*ppe25-pe19* strain a second attenuating mutation, which however preserves ESAT-6 secretion.
(TIF)

S6 Fig Differentiation status of the functional Th1 subsets in mice immunized with a synthetic PPE25 peptide formulated in CpG(DOTAP). Splenocytes from mice immunized with the representative PPE25:1–20 peptide, stimulated with the homologous peptide as detailed in Materials and Methods, stained for the surface differentiation markers, and then by ICS, as described in the Fig 1. Results are means ± SD of experimental duplicates.
(TIF)

S7 Fig T-cell responses specific to PPE25 and PE19 epitopes in BCG-immunized mice. T-cell IFN-γ responses of the splenocytes from C57BL/6 mice (*n* = 4) immunized s.c. with 1 x 10^6^ CFU/mouse of BCG 1173P2 Pasteur strain, at 4 weeks p.i., subsequent to *in vitro* stimulation with PPD, Ag85A:241–260, control MalE:100–114 peptide or individual PPE25- and PE19-derived epitopes. The data are representative of two independent experiments.
(TIF)

S8 Fig CD44 expression and numbers of lung CD4^+^ T cells recovered from immunized mice. A, B) Expression of CD44 activation marker by the lung CD3^+^ CD4^+^ T cells in the immunized or the control mice, as studied *ex vivo* at day 40 in (A) PE/PPE-immunized mice (see Fig 7A) or (B) BCG-primed and PE/PPE-boosted mice, at day 90 (see Fig 8A and 8C) Total numbers of lung CD4^+^ T cells, as determined at day 90 in the BCG-primed and PE/PPE-boosted C57BL/6 mice (*n* = 6 per group), detailed in the legend to the Fig 8. These numbers were determined as total numbers of cells in the Ficoll-treated lung fractions, multiplied by the percentages of CD3^+^ CD4^+^ cells, as assessed by cytometry. NS = not significant, ** or *** = statistically significant, as determined by One Way ANOVA test with Tukey’s correction for multiple comparisons, *p*<0.005 or *p*<0.001, respectively.
(TIF)
