# Supplementary figures and images for: CD4+ T Cells Recognizing PE/PPE Antigens Directly or via Cross Reactivity Are Protective against Pulmonary Mycobacterium tuberculosis Infection
Source: PLoS Pathog. 2016 Jul 28;12(7):e1005770. doi: 10.1371/journal.ppat.1005770 (PMC4965174; doi:10.1371/journal.ppat.1005770)

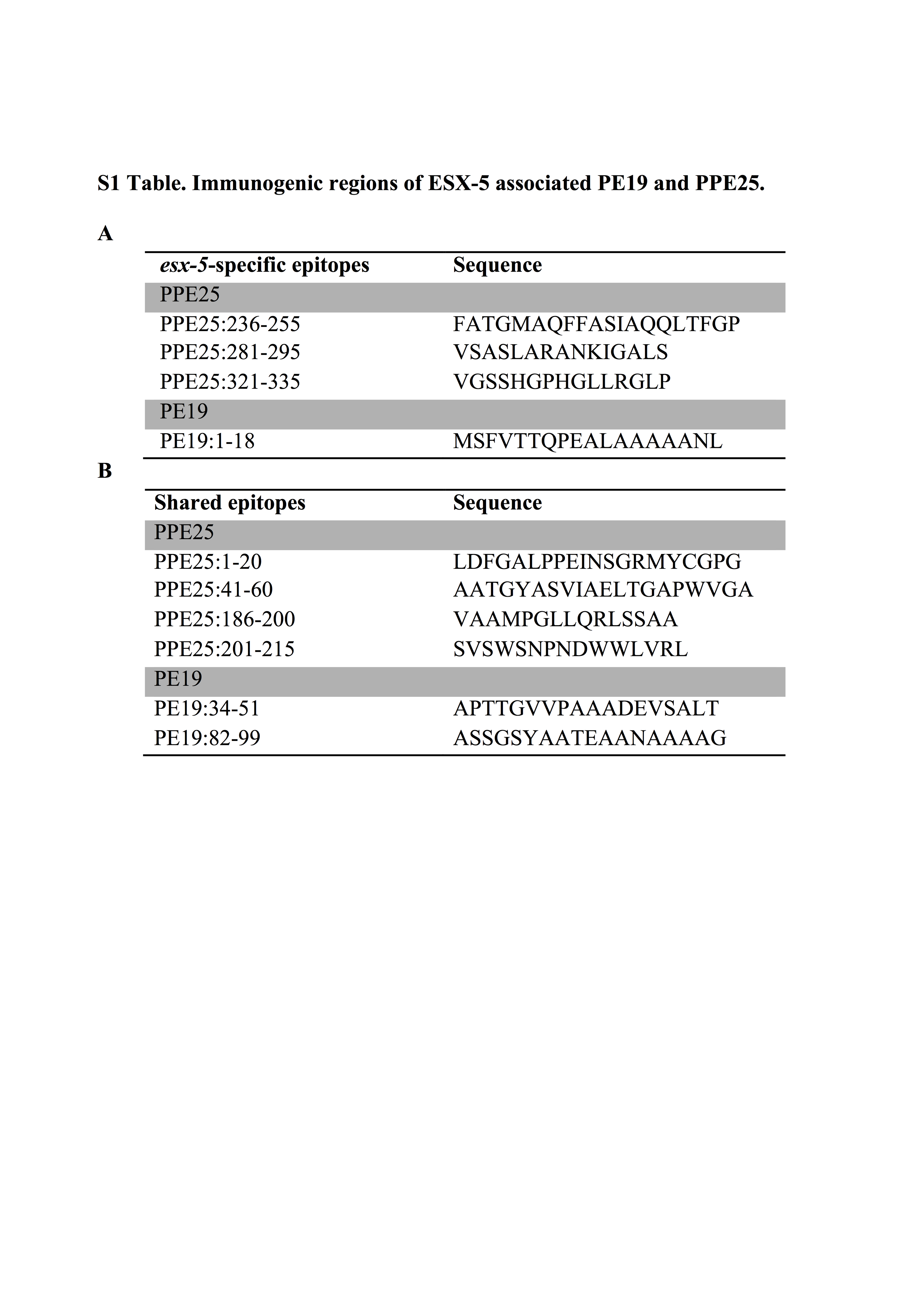

Supplement: S1 Table — PPE25- and PE19-derived peptides, containing MHC-II, I-Ab-restricted T-cell epitopes, either highly specific to Mtb esx-5 region (A) or shared by other PE/PPE homologs coded outside this region (B), as previously identified [5]. (TIF) [file ppat.1005770.s001.tif]

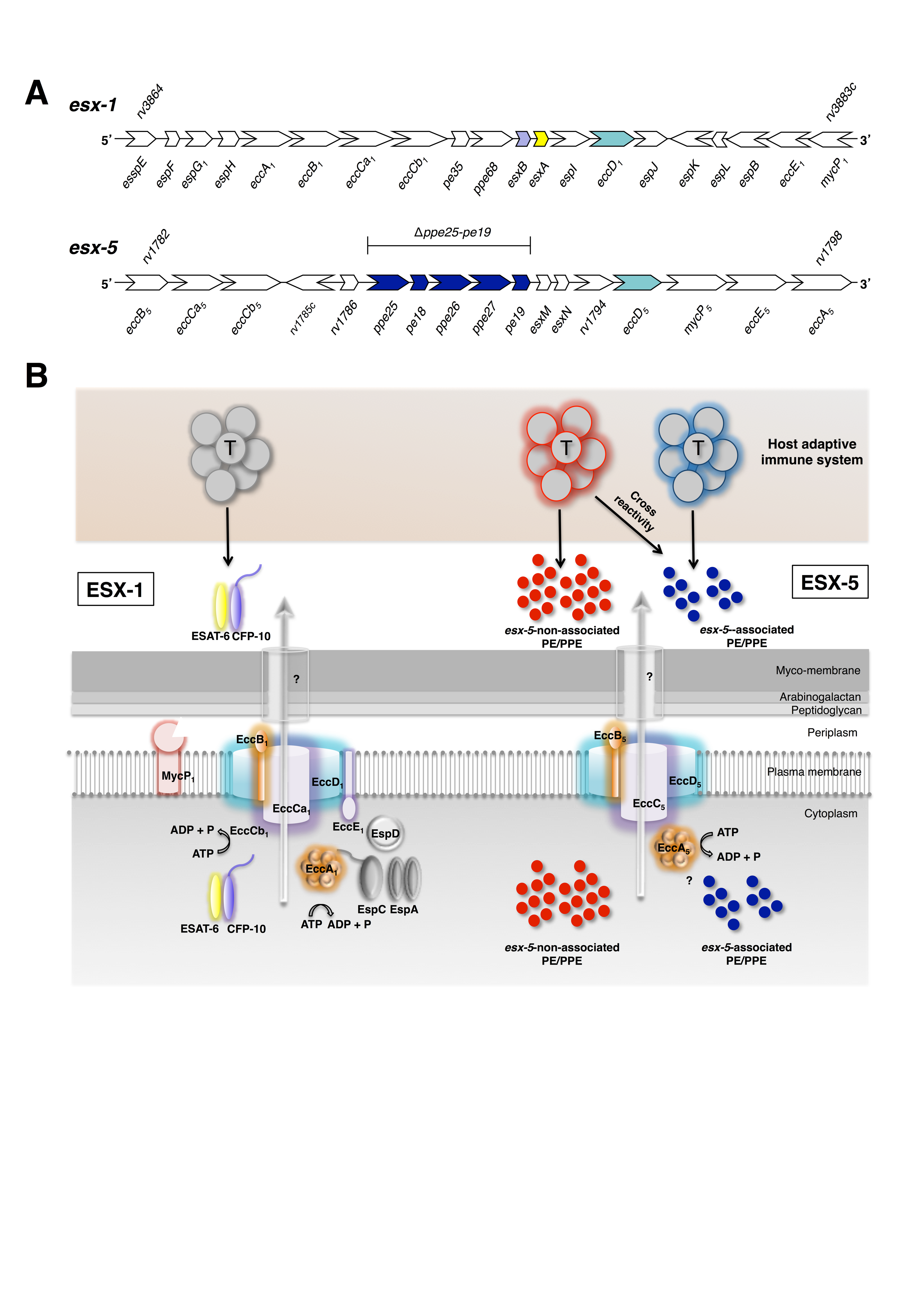

Supplement: S1 Fig — (A) Genetic organization of the esx-1 and esx-5 genomic regions of Mtb. (B) Schematic representation of ESX-1 and ESX-5 T7SSs which export/secret Esx and PE/PPE proteins. Secretion of ESAT-6 and CFP-10 via ESX-1 is responsible of the induction of specific T cells in immunized mice (B, left). Numerous PE/PPE proteins, either coded inside or outside the esx-5 region, are exported/secreted via the trans-membrane EccD5 channel of the ESX-5 system. Subsequent to immunization with the Mtb Δppe25-pe19 strain, secretion of numerous PE/PPE homologs coded outside esx-5 induces T cells, which via cross reactivity, are able to recognize ESX-5-coded PE/PPE virulence-related factors. (TIF) [file ppat.1005770.s002.tif]

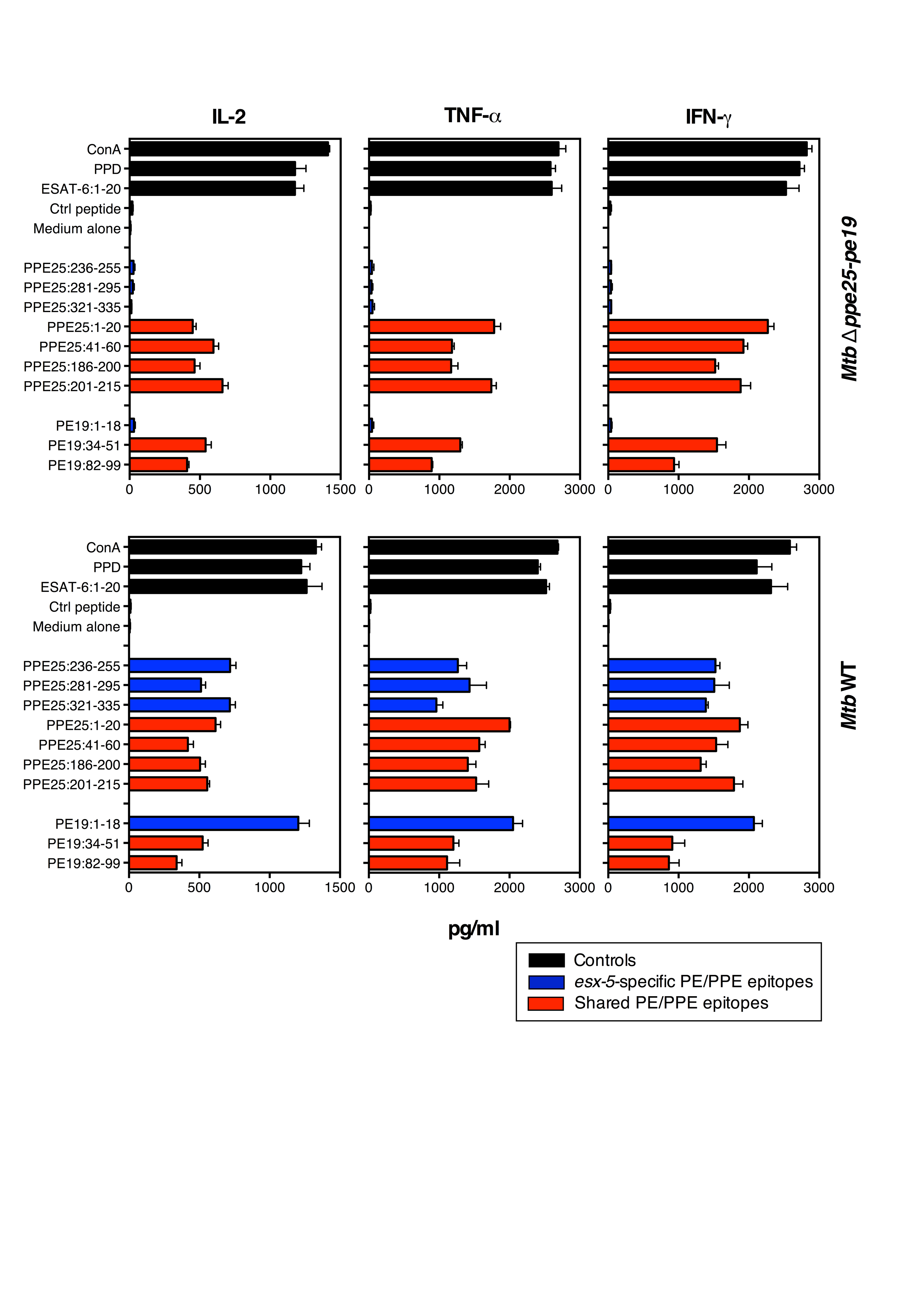

Supplement: S2 Fig — IL-2, TNF-α and IFN-γ production, as quantified by ELISA in the culture supernatants of splenocytes from C57BL/6 mice (n = 5 per group) immunized s.c. with the Mtb Δppe25-pe19 or the Mtb H37Rv WT strain and stimulated in vitro with individual PPE25- and PE19-derived peptides, either highly specific to esx-5 or shared by other PE/PPE proteins coded outside this region at 4 wks p.i. ESAT-6:1–20 or MalE:100–114 peptides were used respectively as positive or negative controls. Error bars represent SD. The results are representative of two independent experiments. (TIF) [file ppat.1005770.s003.tif]

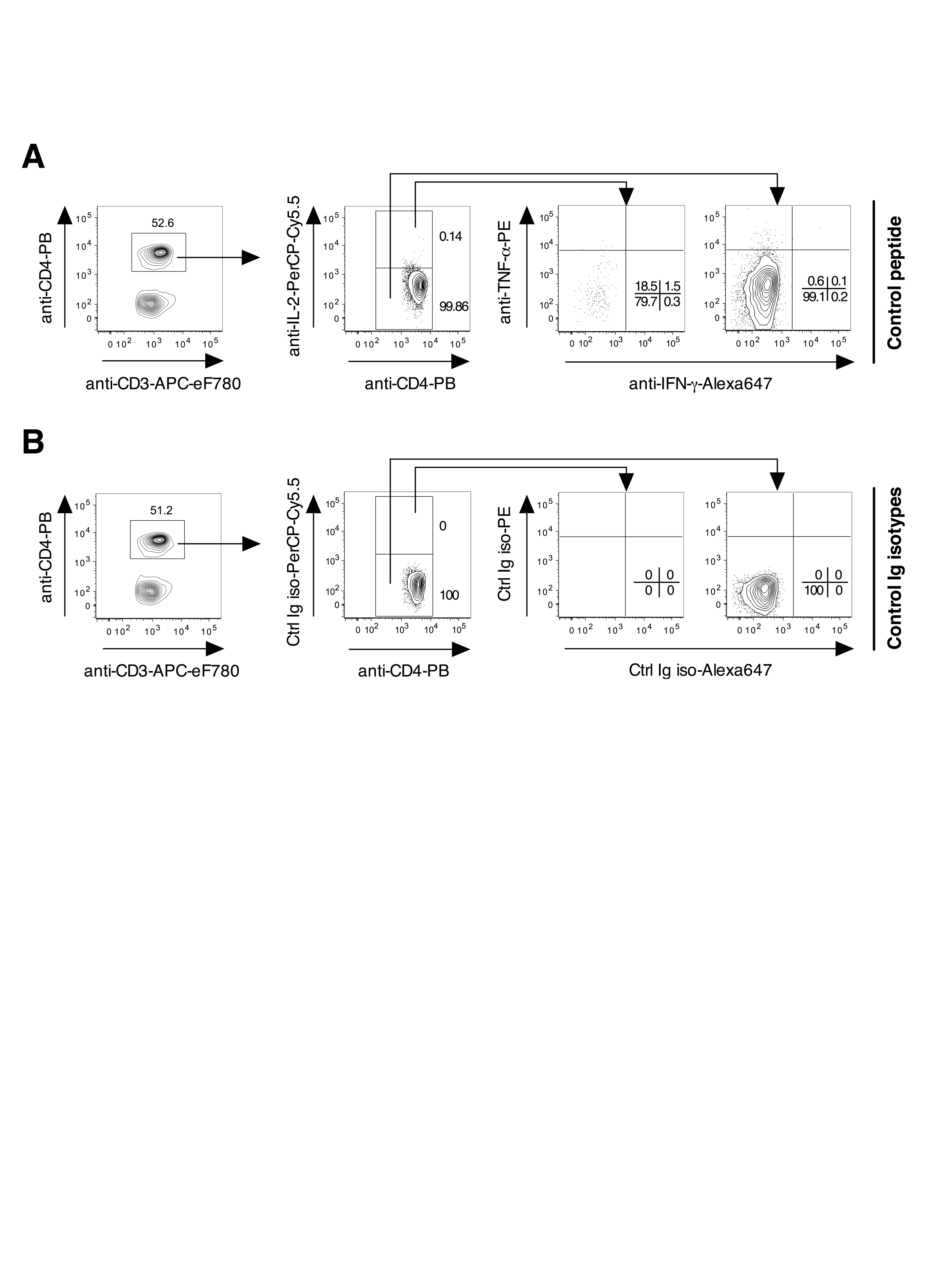

Supplement: S3 Fig — Splenocytes from the Mtb Δppe25-pe19-immunized C57BL/6 mice shown in the Fig 1 were stimulated in vitro with the control MalE:100–114 (A) or homologous peptide (B), prior to surface and intracellular staining with anti-cytokine mAbs (A), or to intracellular staining with control Ig isotypes (B). (TIF) [file ppat.1005770.s004.tif]

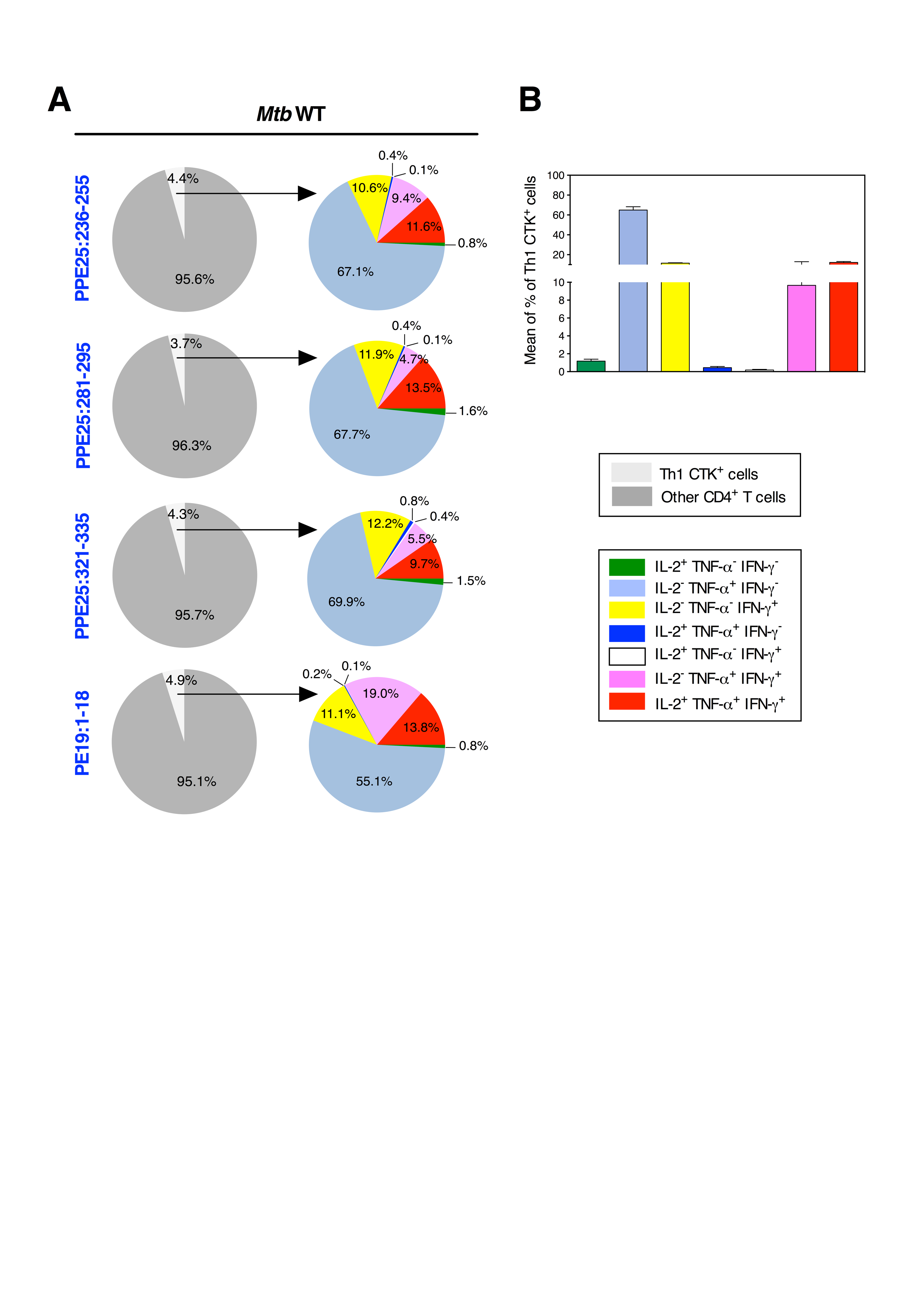

Supplement: S4 Fig — (A-B) Th1 cytokine-producing splenic CD4+ T effectors of C57BL/6 mice (n = 5) at 4 weeks after s.c. injection with 1 x 10^6^ CFU/mouse of Mtb WT strain, analyzed as detailed in the legend to the Fig 1. Such Th1 responses against PE/PPE epitopes specific to esx-5 were not detected in Mtb Δppe25-pe19-immunized mice. (TIF) [file ppat.1005770.s005.tif]

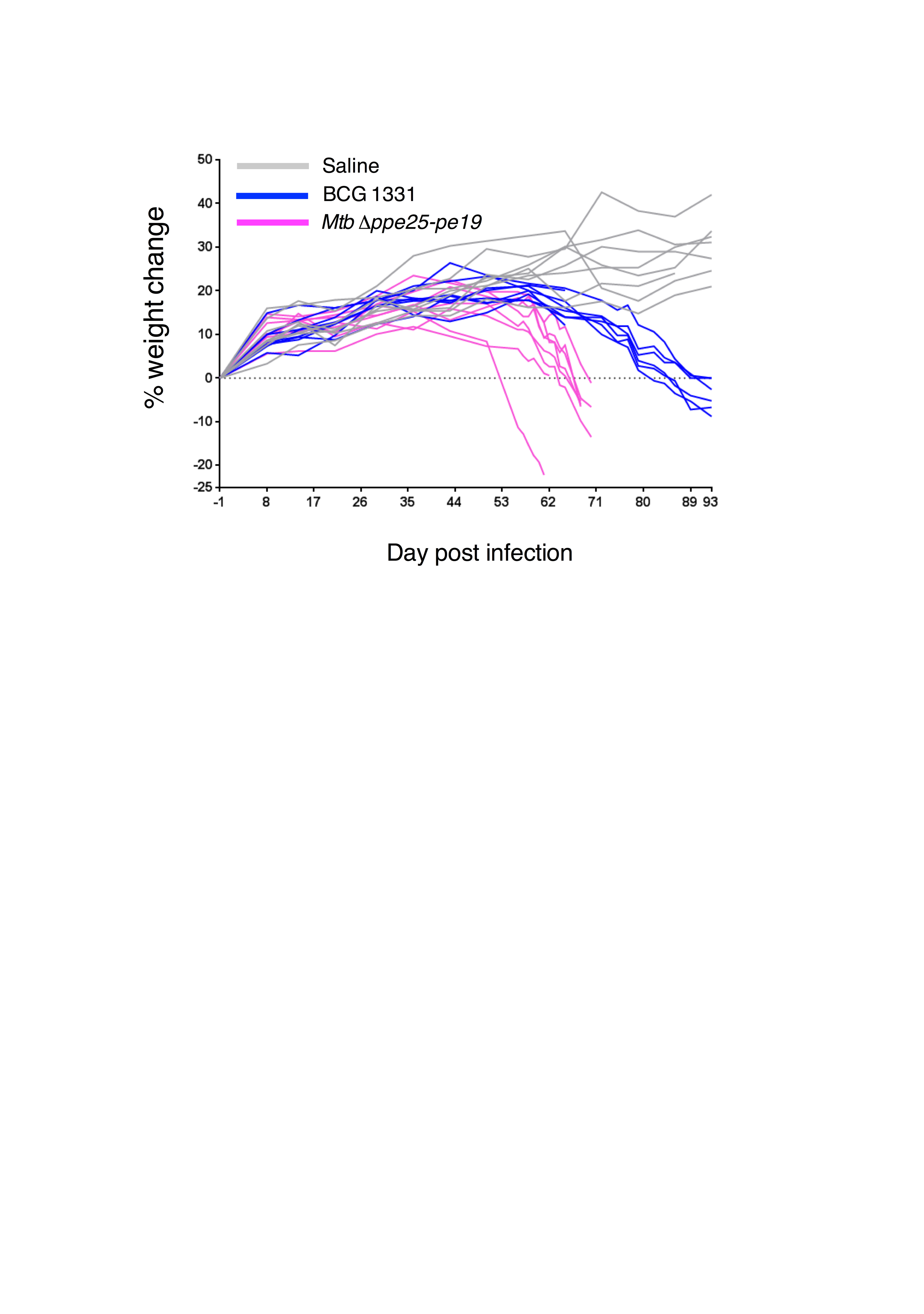

Supplement: S5 Fig — SCID mice were infected i.v. with 1 x 10^6^ CFU/mouse of the BCG Danish 1331 vaccine or the Mtb Δppe25-pe19 strain, as a selected TB vaccine candidate in an independent preclinical virulence trial within the framework of the TBVAC2020 consortium. Control mice received saline only. The weight loss kinetics was followed over a period of 90 days. Animals were euthanized when they reached the human endpoint of >20% weight loss or showed severe clinical signs of disease according to the UK Home Office guidelines referring to the welfare of experimental animals. The Mtb Δppe25-pe19 strain displayed only a weakly higher degree of virulence, as compared BCG Danish 1331, which is the most attenuated live TB vaccine [41, 42]. Work is currently in progress to introduce in the Mtb Δppe25-pe19 strain a second attenuating mutation, which however preserves ESAT-6 secretion. (TIF) [file ppat.1005770.s006.tif]

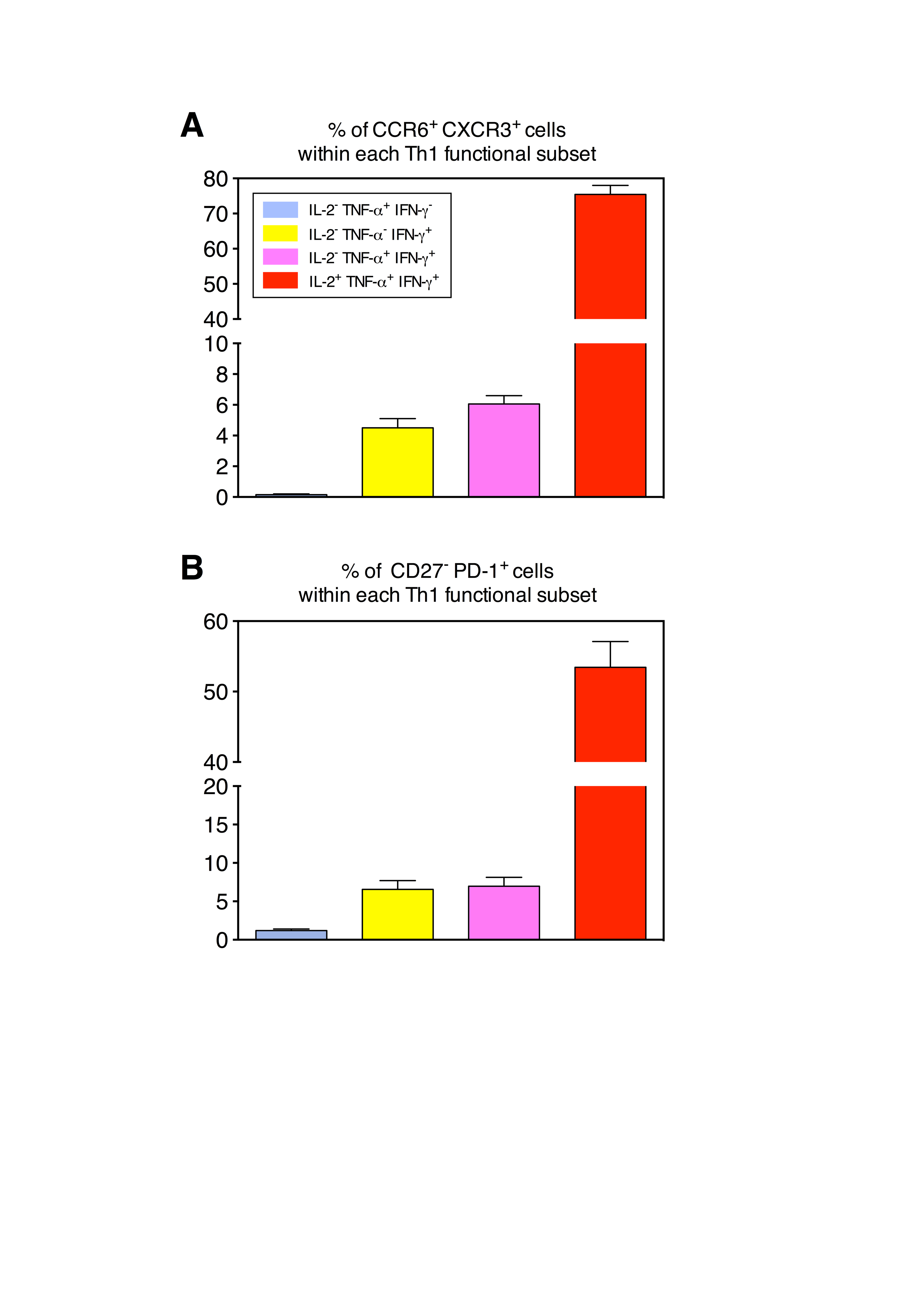

Supplement: S6 Fig — Splenocytes from mice immunized with the representative PPE25:1–20 peptide, stimulated with the homologous peptide as detailed in Materials and Methods, stained for the surface differentiation markers, and then by ICS, as described in the Fig 1. Results are means ± SD of experimental duplicates. (TIF) [file ppat.1005770.s007.tif]

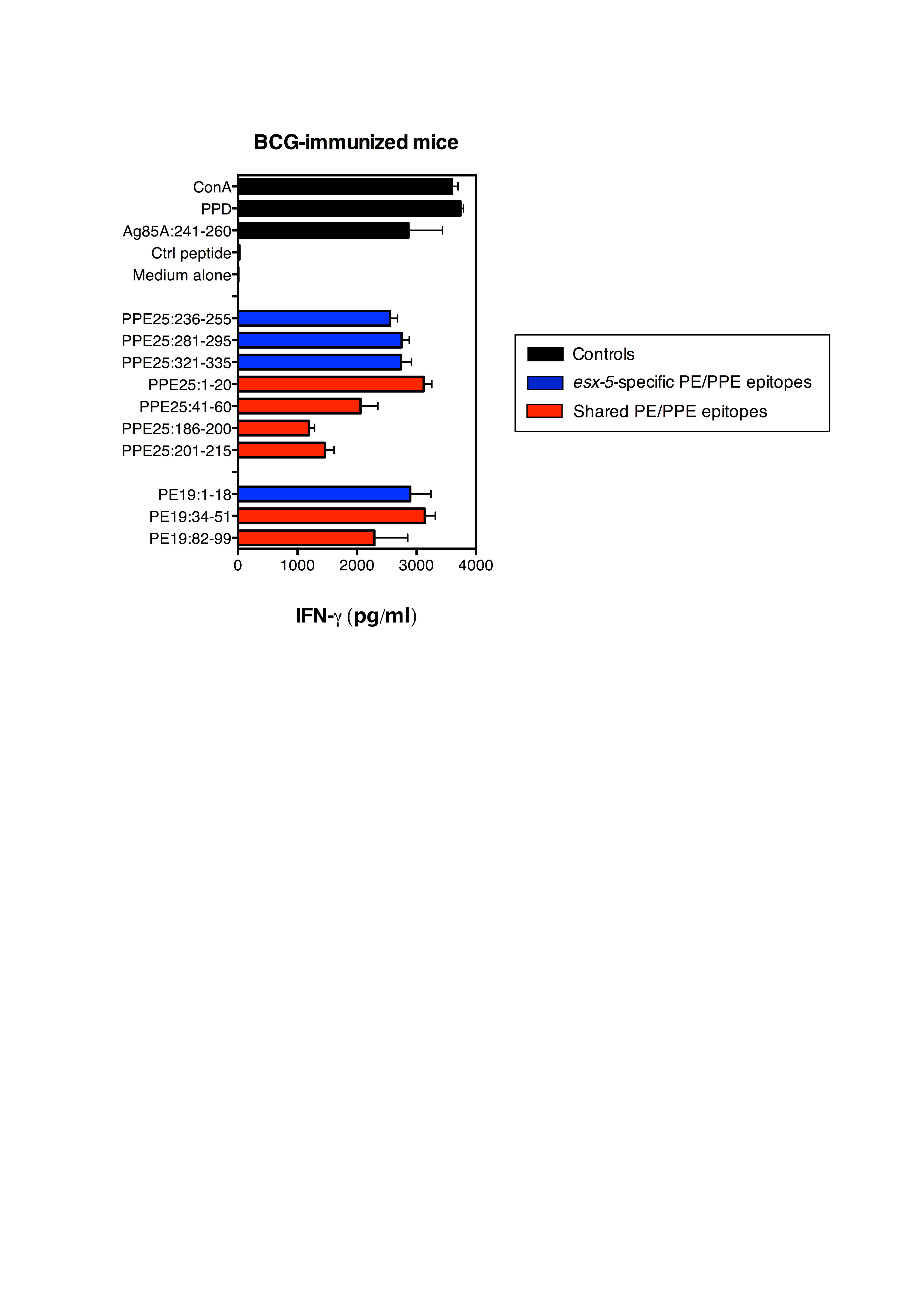

Supplement: S7 Fig — T-cell IFN-γ responses of the splenocytes from C57BL/6 mice (n = 4) immunized s.c. with 1 x 10^6^ CFU/mouse of BCG 1173P2 Pasteur strain, at 4 weeks p.i., subsequent to in vitro stimulation with PPD, Ag85A:241–260, control MalE:100–114 peptide or individual PPE25- and PE19-derived epitopes. The data are representative of two independent experiments. (TIF) [file ppat.1005770.s008.tif]

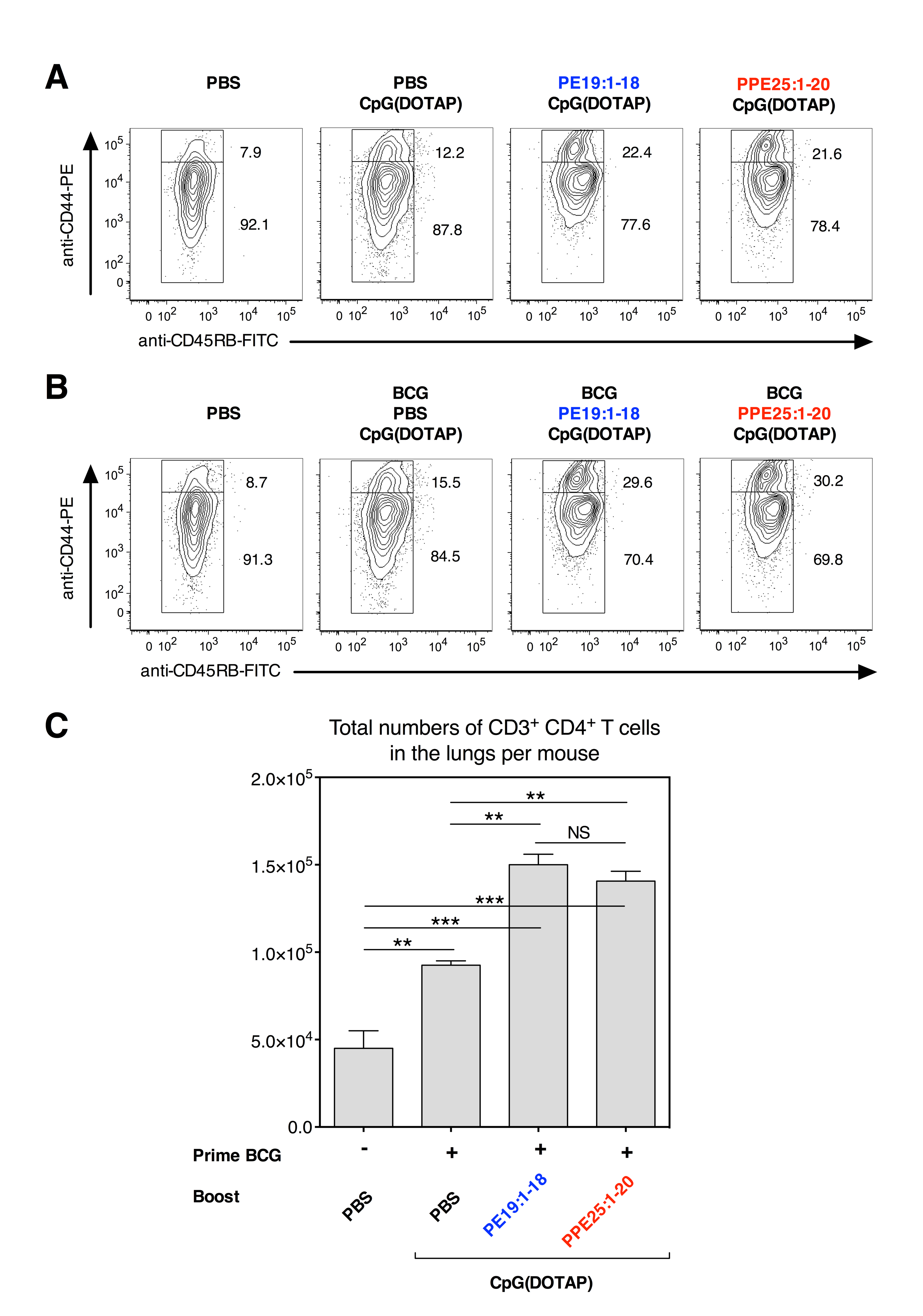

Supplement: S8 Fig — A, B) Expression of CD44 activation marker by the lung CD3+ CD4+ T cells in the immunized or the control mice, as studied ex vivo at day 40 in (A) PE/PPE-immunized mice (see Fig 7A) or (B) BCG-primed and PE/PPE-boosted mice, at day 90 (see Fig 8A and 8C) Total numbers of lung CD4+ T cells, as determined at day 90 in the BCG-primed and PE/PPE-boosted C57BL/6 mice (n = 6 per group), detailed in the legend to the Fig 8. These numbers were determined as total numbers of cells in the Ficoll-treated lung fractions, multiplied by the percentages of CD3+ CD4+ cells, as assessed by cytometry. NS = not significant, ** or *** = statistically significant, as determined by One Way ANOVA test with Tukey’s correction for multiple comparisons, p<0.005 or p<0.001, respectively. (TIF) [file ppat.1005770.s009.tif]
